# Patterns of morbidity, multimorbidity, and mortality in aging cats: findings from seven years of the Cat Prospective Ageing and Welfare Study

**DOI:** 10.3389/fvets.2026.1813450

**Published:** 2026-06-24

**Authors:** Christine R. Pye, Nathalie J. Dowgray, Kelly Eyre, Gina Pinchbeck, David M. Hughes, Delphine Moniot, Eithne Comerford, Alexander J. German

**Affiliations:** 1 Department of Musculoskeletal and Ageing Science, Institute of Life Course and Medical Sciences, University of Liverpool, Liverpool, United Kingdom; 2 Department of Livestock and One Health, Institute of Infection, Veterinary and Ecological Studies, University of Liverpool, Liverpool, United Kingdom; 3 Department of Health Data Science, Institute of Population Health, University of Liverpool, Liverpool, United Kingdom; 4 Royal Canin Research Center, Aimargues, France; 5 Department of Small Animal Clinical Science, School of Veterinary Sciences, University of Liverpool, Liverpool, United Kingdom

**Keywords:** chronic disease, cohort, feline, incidence, lifespan, longitudinal, prevalence, survival

## Abstract

**Introduction:**

Aging is associated with an increased risk of morbidity, multimorbidity and mortality in cats. However, longitudinal cohort studies are somewhat limited in this species. Information about the prevalence and incidence of age-related disease, combinations of morbidities, and factors associated with mortality could provide insights into the impact of aging on the welfare of domestic cats.

**Methods:**

The prevalence and incidence of common age-related diseases, patterns of multimorbidity and causes and risk factors for mortality were investigated in a prospective longitudinal cohort study of 209 aging cats. Cats were enrolled at middle age and attended a veterinary examination every 6 months, until death or censoring, for up to 7 years.

**Results:**

A total of 1,241 examinations were performed (median follow-up 4.4 years; range 0.0–6.9). Dental disease (84%), abnormalities on orthopedic examination (82%) and heart murmurs (59%) were most prevalent within the cohort over the study period. Most cats (96%) developed at least one abnormality on veterinary examination, and multimorbidity was common, with 175 cats (84%) developing two or more morbidities during the study. Death occurred in 59/209 cats (28%) and median life expectancy was 15.2 years. Pedigree cats had approximately double the hazard of mortality (HR 2.62, 95%-CI 1.39, 4.93; *P* = 0.003) compared with mixed-breed cats, with a similar increase in hazard for those with muscle loss at middle age (HR 2.01, 95%-CI 1.13, 3.60, *P* = 0.018) compared with cats without muscle loss.

**Discussion:**

These findings demonstrate that age-related morbidity and multimorbidity are highly prevalent in cats and both should be considered by veterinary professionals examining these older animals. Furthermore, we identified that midlife body condition, muscle condition and pedigree status affected mortality risk, highlighting the importance of assessing these factors in practice and suggesting that targeted strategies to promote healthy aging should start from middle age.

## 1 Introduction

There are an estimated 11 million owned cats in the UK and 61 million owned cats in the USA, with approximately 26% of households in both countries thought to own a cat (1, 2). The average life expectancy for cats attending primary care veterinary practices in the UK is estimated to be 14 years (3). Certain diseases (e.g. chronic kidney disease [CKD], hyperthyroidism, diabetes mellitus, cardiovascular disease, hypertension, degenerative joint disease and neurological disorders) have an increased prevalence in older cats (4–6). These age-related diseases can adversely impact a cat's quality of life, and early diagnosis and interventions could help to reduce this impact (7–9).

The underlying biology of aging is complex, involving many cellular and molecular mechanisms that affect multiple organ systems (10). Aging is associated with the accumulation of multiple chronic conditions within the same individual. Although the terms comorbidity and multimorbidity are sometimes used interchangeably, comorbidity traditionally refers to additional diseases in relation to an index condition, whereas multimorbidity considers co-existing chronic conditions without prioritizing a single disease process (11). Aging cats commonly present with several concurrent age-related disorders (12, 13), which can present a challenge in veterinary practice. Longitudinal studies are important to understand better the development of multimorbidity, including the accumulation of chronic disorders over time, patterns of disease co-occurrence, and their effects on survival.

Mature and senior cats are at increased risk of developing chronic age-related diseases, making regular health assessment an important component of preventive veterinary care (4, 8, 14). Current American Animal Hospital Association (AAHA) and Feline Veterinary Medical Association (Feline VMA) guidelines classify mature (middle aged) cats as between seven and 10 years old, and senior cats as over 10 years old (Quimby et al., 2021). Improved understanding of morbidity and multimorbidity trajectories in aging cats could support earlier diagnosis, monitoring, and management of age-related diseases, leading to improved cat welfare and better support for pet owners.

Existing evidence on aging-related morbidity in cats is limited primarily to cross-sectional studies or investigations of individual diseases. Large scale retrospective studies using electronic health record data from primary care veterinary practices have provided valuable information on prevalence of age-related disease, causes of mortality and breed-related differences in life expectancy in cats (15, 16, 97). However, their cross-sectional design does not allow for incidence rates of disease to be calculated. Longitudinal cohort studies have focused on single disease processes (17–21), have a relatively short follow-up period (9), or have not yet reported on the effects of aging in mature to senior cats (22). There is, therefore, limited evidence describing long-term morbidity, multimorbidity, and mortality outcomes in longitudinal cohorts of aging client-owned cats. Improved knowledge of the development of morbidity and multimorbidity from such a study could inform veterinary wellness plans, improve advice for preventive care and, ultimately, improve understanding of the aging process in this species.

The Cat Prospective Ageing and Welfare Study (CatPAWS) was established in 2017 with the aim of following a cohort of >200 client-owned pet cats from middle age to end-of-life. The clinical findings at enrolment in 206 cats from this cohort have been previously reported (8), and findings from an initial 2-year follow-up period were described in a doctoral thesis (23). The current study reports longer-term outcomes related to disease incidence, multimorbidity, and mortality in this ongoing cohort, with follow-up periods of up to 7 years.

The aims of this study were: (1) to estimate the prevalence and incidence of common age-related diseases during longitudinal follow-up of a cohort of aging pet cats; (2) to characterize patterns of multimorbidity over time; and (3) to describe mortality outcomes, including causes of death, survival time, and clinical variables associated with increased mortality risk.

## 2 Materials and methods

### 2.1 Ethical approval

Ethical approval for this study was granted by the University of Liverpool Veterinary Research Ethics Committee, under the code VREC491 (with amendments a, b, c, d and e), and the Royal Canin Ethical Review Committee. All owners gave their informed consent, in writing, before their cat was enrolled in the study.

### 2.2 Data collection

Data were collected from cats enrolled in CatPAWS between February 2017 to January 2024; complete study methodology for CatPAWS (including enrolment criteria, study design and clinical information) has been published elsewhere (8, 23), and is also summarized in the supplementary information (Supplementary Figure 1). Briefly, most cats were enrolled between 7 and 10 years of age, with eight cats being marginally outside this age range at enrolment. All cats underwent a veterinary examination every 6 months until death or censoring at the census date (January 2024). At each examination, bodyweight was measured using portable V20 feline scales (Burtons, UK), which were regularly calibrated with a “test” weight, whilst body condition score (BCS) was assessed using a 9-unit scale, as previously described (Laflamme, 1997). Muscle condition score (MCS) was assessed at 10 separate skeletal landmarks: skull, neck, thoracic vertebrae, lumbar vertebrae, left and right scapula, left and right gluteal muscle group and left and right hindlimb muscle groups caudal to the femur (24). Each skeletal landmark was graded from 0 (severe muscle wastage) to 3 (no muscle wastage) using a validated scale (Michel et al., 2011), with a combined MCS score up to a maximum of 30 points created by summing results from all 10 landmarks (24). Systolic blood pressure (SBP) measurements were taken by the Doppler technique with either a Vet BP Doppler (Burtons) or a CAT+ Doppler (Thames medical) blood pressure monitor, and a 2.5 to 3 cm cuff. Measurements of SBP complied with standard guidelines, usually made from the right or left palmar common digital artery, or from the tail coccygeal artery (25). Five readings were obtained and the average of these taken.

A complete physical examination was performed, including cardiac and lung auscultation, abdominal palpation, assessment of eyes, nose, oral examination, aural examination and coat and skin assessment. Cardiac auscultation was completed up to three times per clinical examination, noting the heart rate, the presence of any arrhythmias, and the presence and intensity of any heart murmurs graded from one to six (26–28). Blood samples were collected, using either jugular or cephalic venepuncture, and analyzed for routine hematology and serum biochemistry panels, as per the methods outlined by Dowgray et al. (8). Urine samples were obtained, either by “free catch” or by cystocentesis where practicable, and urinalysis was performed as per the methods outlined by Dowgray et al. (8).

### 2.3 Criteria for diagnosis of disease

Criteria for diagnosing specific morbidities have been previously described (8, 23) and are summarized below.

Chronic kidney disease (CKD) was diagnosed when plasma creatinine concentration was measured above the reference interval (>177 μmol/L) in at least two blood samples, in the absence of identifiable pre-renal causes, ideally with accompanying urinalysis demonstrating impaired urine concentrating ability (urine specific gravity [USG] < 1.035; (17, 18)). Where contemporaneous urinalysis demonstrated a USG < 1.035 together with an increasing plasma creatinine concentration over the study period, progressing to ≥140 μmol/L in at least two blood samples and in the absence of identifiable pre-renal causes, this was also considered to meet the diagnostic threshold, consistent with International Renal Interest Society (IRIS) classification of at least stage II CKD (98). During the study period, plasma creatinine concentrations were measured by two different laboratory analysers, which had upper reference intervals of 177 and 212 μmol/L, respectively. To maintain consistency in CKD case definition across the study and to minimize potential bias related to analyser-specific reference intervals, a single upper reference limit of 177 μmol/L was applied throughout to ensure uniform classification of CKD status over time.

Hyperthyroidism was diagnosed when the plasma total thyroxine concentration was ≥60 nmol/L, or >50 nmol/L with an increased free thyroxine concentration confirming hyperthyroidism (29). Hypertension was diagnosed when the SBP was consistently ≥160 mmHg on subsequent SBP measurements (25). Diabetes mellitus was diagnosed when persistent hyperglycaemia and glucosuria were present with consistent clinical signs, and stress-induced hyperglycaemia had been excluded (30).

An oral examination was undertaken with each quadrant of dentition being graded for both gingivitis and calculus using a scoring system of 0 to 4. The presence of feline odontoclastic resorptive lesions (FORLS) was recorded, as was the identification of fractured teeth, oral lesions or stomatitis. Dental disease was diagnosed if a cat had a gingivitis score of ≥2, or any FORLs or stomatitis, equivalent to the point when veterinary advice of a dental treatment under general anesthetic was provided (8, 21). For the current study, the term neoplasia encompassed multiple neoplasia types, including those diagnosed on physical examination alone (*e.g*. oral neoplasia, palpable abdominal masses) or after further diagnostic investigations such as imaging and histology *e.g.*, lymphoma, malignant mammary carcinoma, renal carcinoma (20, 31).

A veterinarian (CP or ND) conducted a complete orthopedic examination (OE) in amenable cats using the methodology previously reported (32, 33). For those cats where a full OE was completed, each limb was assigned a score of 0 (no abnormalities in any joint), 1 (either reduced range of movement (ROM) or pain in one or more joints), 2 (reduced ROM and pain detected or palpable thickening of a joint or joint effusion detected) or 3 (reduced ROM and pain and palpable joint thickening in one or more joints). Coxofemoral joints were separately scored as 0 (no abnormalities in either coxofemoral joint), 1 (either pain or reduced ROM in one or both coxofemoral joints) or 2 (pain and reduced ROM in one or both coxofemoral joints). A total OE score from 0 to 14 was assigned for each complete OE, with a larger score indicating greater musculoskeletal impairment (23).

### 2.4 Multimorbidity Score

As previously determined by Dowgray (23), a multimorbidity score of 0 to 8 was assigned to cats at each examination, whenever diagnostic information on the following conditions was available. The following conditions or clinical examination abnormalities recorded in this score included: CKD, neoplasia, hypertension, hyperthyroidism, heart murmur, diabetes mellitus, dental disease and any degree of orthopedic examination abnormalities.

### 2.5 Statistical analysis

Unless otherwise stated, all statistical analysis was undertaken using the open-access statistical language and environment, R version 4.4.1 (34). The additional packages “dplyr” version 1.1.4 (35), “ggplot2” version 3.5.1 (36), “sjPlot” version 2.8.16 (37), “gtsummary” version 1.7.2 (38) and “purr” version 1.0.4 (39) were used to examine, manipulate and visualize the data. Additional packages relating to specific statistical analyses are given in the relevant sections below. Continuous data were assessed for normality using histograms, quantile-quantile plots and Shapiro-Wilk testing and described using either mean and standard deviation (SD) or median and interquartile range (IQR), depending on whether or not their distribution was gaussian.

#### 2.5.1 Sample size calculation

A sample size calculation was performed prior to enrolment and estimated a cohort of 194 cats would be required for a cohort study investigating incidence of disease or mortality. This calculation was undertaken using an online tool (https://epitools.ausvet.com.au/) and assumed a 95% confidence level, a desired power of 80%, an expected incidence of 10% in the unexposed group and a relative risk of 2.5% (for exposures of 20%; (23)).

#### 2.5.2 Prevalence and incidence rates

Prevalence was reported as the number (percentage) of cats with a particular morbidity, both as prevalence during the entire study period and prevalence in cats by age year group, including pre-existing cases and cases diagnosed at enrolment. Each cat was counted once per age-year group. The prevalence estimates by age-year group and sex were descriptive rather than inferential, with the aim of describing the disease burden across age and sex groups in the cohort. For each disease, 95% confidence intervals (95%-CI) for prevalence were calculated using the Wilson score interval (40).

Binomial mixed-effects logistic regression models testing associations of age, sex and the interaction of age and sex with the diagnosis of each morbidity were explored to account for repeated observations within cats. Models were fit using the *glmer* function of the “lme4” package version 1.1-37 (41) and “splines” package version 4.4.1 (34). In these models, the dependent variable was a binary variable of whether that condition was diagnosed (yes vs. no), fixed effects were age modeled as continuous variable (in decimalised years) with natural (restricted) cubic splines at three degrees of freedom to allow for non-linear age-related changes, sex (binary male vs. female), and the interactions of age (modeled using natural cubic splines at three degrees of freedom) and sex. For several conditions (*e.g*. diabetes mellitus, CKD, neoplasia and hyperthyroidism), these models failed to converge or produced unstable estimates, due to limited event counts. Conditions with sufficient sample size (hypertension, heart murmurs, OE abnormalities and dental disease) were presented as plots of the predicted probability of disease presence using “sjPlot” and “ggplot2”. Likelihood ratio tests (LRT) were applied to investigate whether age and sex significantly improved model fit compared to models without these separate variables. Significance was set at *P* < 0.05. Models were assessed for convergence, inspection of simulated residuals and evaluation of overdispersion using the “DHARMa” package (42), and posterior predictive check using the “performance” package (43).

Incidence per cat-year at risk was calculated by dividing the number of new cases of disease (excluding cases already diagnosed at enrolment from the numerator and denominator of this calculation) by total cat-time at risk in days (which is the time from enrolment to diagnosis, death or loss to follow up or study end date) and dividing by 365.25 to account for leap years (44).

#### 2.5.3 Age at diagnosis for conditions not present at enrolment

The age at diagnosis was recorded as either the age when the diagnosis was made, or the age at the first study examination following a diagnosis made at another veterinary practice when clinical records from that practice were unavailable. Cats with pre-existing conditions at the time of enrolment to the study were excluded from this specific analysis due to the age of diagnosis being unknown if made prior to enrolment. Therefore, these analyses were examining the age at diagnosis where that condition was not present at the enrolment examination. Information on disease status at enrolment to the study has been previously published (8).

#### 2.5.4 Analysis of multimorbidity

Associations with the following morbidities and clinical findings were assessed: diabetes mellitus, hyperthyroidism, neoplasia, CKD, hypertension, heart murmurs (of any grade), OE abnormalities (of any grade) and dental disease. Firstly, the sample sizes of different combinations of morbidities that occurred in the study cats were calculated. Secondly, pairings of morbidities diagnosed by the end of the study period in the cats were calculated. Thirdly, correlation analyses amongst different morbidities, and also with mortality, were explored using the Phi coefficient (which assesses associations between binary variables), and results were displayed using a heatmap created with the “corrplot” package [version 0.92; (99)]. The Phi coefficient, mathematically equivalent to Pearson's correlation for binary data, was calculated using Pearson's method applied to the binary dataset. Corresponding *P*-values were computed and then adjusted for false discovery rate (FDR) using the Benjamini-Hochberg method. Significance was set as FDR adjusted *P* < 0.05. In this correlation analysis the following morbidities were included as binary variables: heart murmur, hypertension, hyperthyroidism, diabetes mellitus, CKD, neoplasia, dental disease, muscle loss of moderate-to-severe (combined MCS 0-19/30), OE score of ≥5. Given the high prevalence of OE abnormalities and muscle loss over the study period, only cats with an OE score of ≥5 at any timepoint [classified as “severe”; (23)] and muscle loss of moderate-to-severe (MCS 0-19/30), were included when determining associations with other morbidities to retain meaningful variability.

Finally, a Poisson generalized linear mixed-effects model was created using the *glmer* function of the “lme4” package to assess associations between cat age and changes in multimorbidity scores. In this random intercept model, age in decimalised years was the sole fixed effect and the individual cat was included as a random effect to enable data from multiple visits to be included. Model fit was assessed using the “DHARMa” residual diagnostics package version 0.4.7 (42); no evidence of overdispersion or zero-inflation was found.

#### 2.5.5 Mortality analysis

Survival analyses were performed using the “survival” package version 3.7.0 (45) within R. The primary aim of the survival analyses was to identify baseline risk factors associated with mortality at enrolment to the study in the cats.

Age (in decimal years, calculated as days divided by 365.25 to account for leap years) was used as the underlying time scale. Survival time was defined as the age at death or the age at censoring (*i.e*. when the cat was still alive at the end of the study or lost to follow-up). Survival status was recorded as a binary variable indicating whether the cat had died (event) or was censored. Statistical significance was set at *P* < 0.05.

To ensure consistency and comparability across models, univariable and multivariable Cox proportional hazards regression analyses were performed, using a complete-case dataset and including only cats with all the following predictor variables recorded at enrolment. Predictor variables tested were: BCS (categorized as 3–4, 5, 6 and 7–9 with BCS 5 as the reference category; no cat had a BCS < 3 at enrolment), MCS (categorized as no muscle loss [combined MCS 30/30] and mild muscle loss [combined MCS 20–29/30; 24]; no cat had a combined MCS < 20 at enrolment); bodyweight (kg), sex (male vs. female), breed (pedigree vs. non-pedigree), binary indicators of disease at enrolment including CKD, hypertension, hyperthyroidism and dental disease, and the presence of a heart murmur (categorized as none, grades 1–2, or ≥3). Neoplasia and diabetes mellitus at enrolment were not included because only a single cat was affected for each, and these cats were excluded from these analyses.

A separate univariable Cox model, investigating association of multimorbidity score at enrolment with mortality, was applied to a reduced dataset due to missing data. Multimorbidity scores were categorized as 0, 1, 2, 3 or 4–5 morbidities due to only two cats having a multimorbidity score of 5 and none with a multimorbidity score >5 at enrolment.

First, analyses of separate predictor variables at enrolment on risk of death was completed using univariable Cox proportional hazards models. The proportional hazards assumption was assessed using scaled Schoenfeld residuals and *Z*-tests (using the function *cox.zph* in the “survival” package).

Where violations of the proportional hazards assumption were identified in univariable analyses (as occurred for BCS categories), a time-dependent Cox proportional hazards model was also fit, by splitting survival time using the *survSplit* function of the “survival” package. An initial cutpoint at 10 years of age was considered based on feline mature-senior life stage classifications (Quimby et al., 2021). Examination of Schoenfeld residuals suggested a change in the hazard relationship around 11 years, and models using an 11-year split provided improved proportional hazards diagnostics and more stable estimates. Therefore, the 11-year split model is presented.

Multivariable Cox proportional hazards regression was then used to identify independent predictors of mortality. The multimorbidity score was not included in the multivariable model because it is a composite variable directly derived from the individual morbidities that were evaluated as separate candidate predictors. Exhaustive subset selection was performed as an exploratory approach to evaluate all candidate predictors; all possible combinations of the 10 predictor variables (1,023 models) were evaluated. Model performance was compared using Akaike's Information Criterion (AIC) and Bayesian Information Criterion (BIC); however, model selection was ultimately based on BIC to prioritize parsimony given the relatively limited number of events. The final model had the smallest BIC and met the proportional hazards assumption.

Kaplan Meier curves and Cumulative Hazards curves were assessed visually using “survival”, “ggsurvfit” version 1.1.0 (46) and “survminer” version 0.4.9 (47) packages in R.

Internal validation of the final Cox model was performed using bootstrap resampling (1,000 iterations) to estimate optimism-corrected performance, implemented using the *validate* function of the “rms” package version 8.1–0 (48).

## 3 Results

### 3.1 Sample size and summary data

A total of 209 cats, belonging to 149 owners, were enrolled between 7th February 2017 and 5th March 2020. Data from the enrolment examinations of 206 of these cats have been published previously (8), and body condition metrics for these cats have also been recently reported (24). Clinical data from enrolled cats were collected up to 25th January 2024 and included a total of 1,241 visits, with a median of six visits per cat (IQR 3, 9; range 1, 12). Twenty-nine cats (14%) only attended for the initial visit, whilst the remaining 180 cats (86%) attended for ≥2 visits. The median follow up time was 4.4 years (IQR 1.8, 5.7; range 0.0, 6.9).

A summary of the signalment and visit data for the 209 cats is included in the supplementary information (Supplementary Tables 1, 2). There were 111 (53%) female cats and 98 (47%) male cats, only two of which were sexually intact (both male cats). One hundred and eighty cats (86%) were non-pedigree mixed breed, with the remaining 29 cats (14%) representing 14 different pedigree breeds. Eight cats were marginally outside the target recruitment age at enrolment (seven cats were marginally younger [aged 6.7–7.0 years] and one cat marginally older [aged 11.4 years]) either because of inaccurate age records held by the primary veterinary practice, or because of inaccurate owner recall at the time of recruitment (23). Therefore, the median age at enrolment was 8.1 years (IQR 7.2, 9.3 years). The median age of the cohort across all study visits was 10.3 years (IQR 8.8, 12.0 years; range 6.7, 16.4 years).

### 3.2 Loss to follow up

During the study, 59 cats (28%) were lost to follow up, and 59 cats (28%) died, with median time before withdrawal being 443 days (IQR 1.5, 686.5 days; range 1, 2,415 days). The most common reason for withdrawal was lack of owner response to communications from clinical staff (*e.g.*, booking appointments; 28, 48%), followed by owners moving house (9, 15%) and owners withdrawing due to their cat being too stressed by visits (7, 12%; Supplementary Table 3).

### 3.3 Prevalence and incidence rates of age-related morbidities

**Figure 1:**
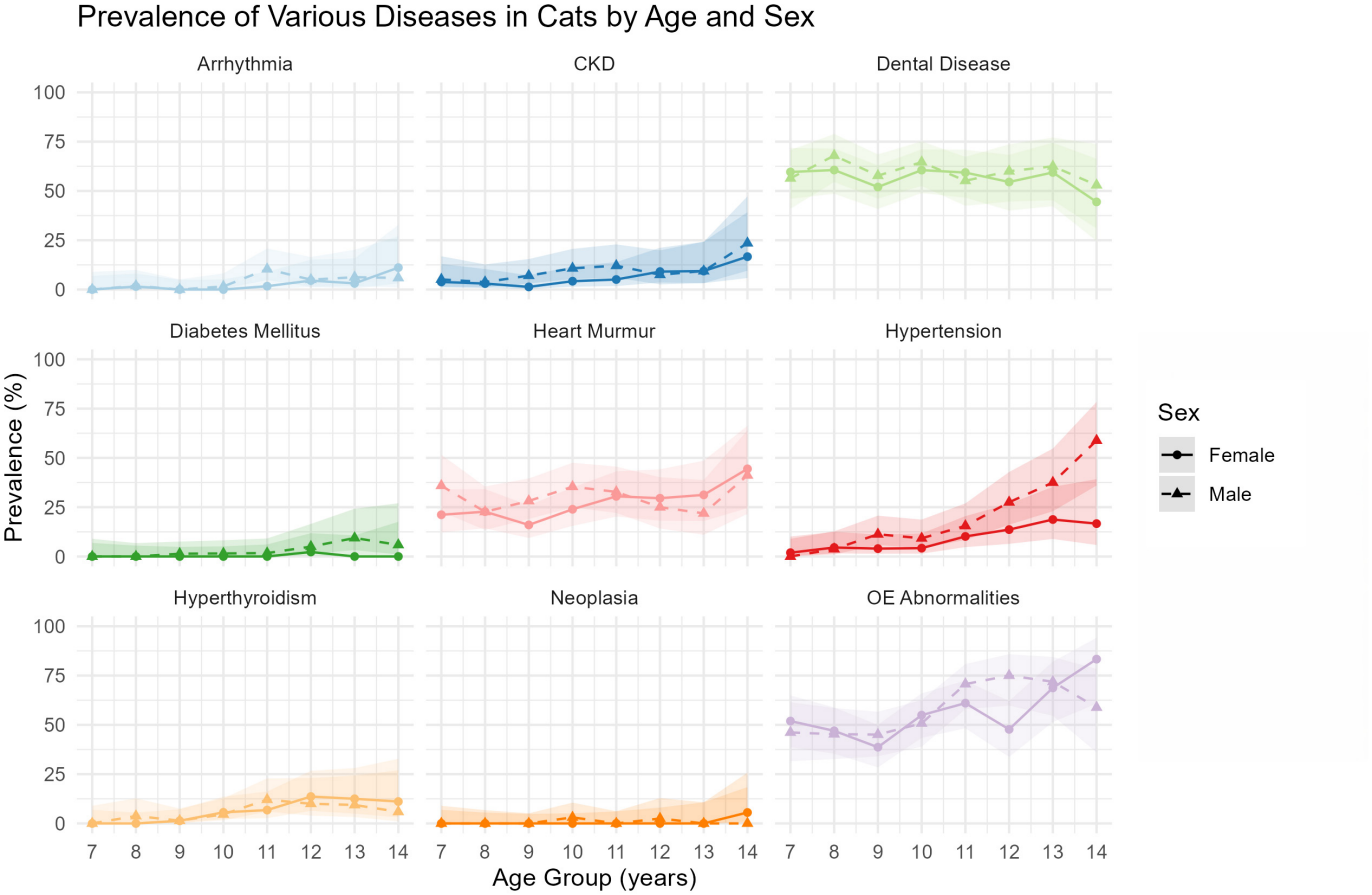
Observed age-specific prevalence of selected diseases in the 209 study cats, stratified by sex. Number of cats at each year of age by sex were as follows: age 7 year: 52 female, 39 male; age 8 year: 66 female, 53 male; age 9 year: 75 female, 71 male; age 10 year: 71 female, 65 male; age 11 year: 59 female, 58 male; age 12 year: 44 female, 40 male; age 13 year: 32 female, 32 male; age 14 year: 18 female, 17 male. Shaded ribbons around the lines represent 95% confidence intervals. If a cat had more than one veterinary examination within the year, only data from one examination (prioritizing the visit with diagnosis of disease) was included to avoid duplicates of the same cat per year group. Sample sizes declined at older ages due to staggered enrolment, withdrawal from follow-up, and mortality. These estimates are descriptive and do not account for repeated measures contributed by individual cats across multiple age groups. CKD, chronic kidney disease; OE, orthopedic examination.

Prevalence and incidence rates of certain age-related morbidities in the cats over the course of the study period are shown in Table 1. The most prevalent conditions were dental disease (84%, 95%-CI 79, 89%), OE abnormalities (82%, 95%-CI 77, 87%) and heart murmurs (59%, 95%-CI 53, 66%). The descriptive prevalence of conditions per age-year group by sex is shown in Figure 1, with the prevalence per age-year group over all cats (not stratified by sex) shown in Supplementary Figure 2.

**Table 1:** Prevalence, incidence rates and age at diagnosis for various diseases in the 209 study cats.

Disease	Number of cats^1^	Number at enrolment^2^	Incident cases^3^	Time at risk^4^	Prevalence^5^	Incidence rate^6^	Age at diagnosis^7^
Dental disease	176	129	47	118	84.2 (78.7, 88.5)	0.398 (0.284, 0.512)	9.3 (8.3, 10.3)
OE abnormalities	172	130	42	85	82.3 (76.6, 86.9)	0.375 (0.245, 0.505)	10.1(9.1, 10.6)
Heart murmur	124	61	63	407	59.3 (52.6, 65.8)	0.155 (0.117, 0.193)	10.4(9.3, 11.6)
Hypertension	41	6	35	668	19.6 (14.8, 25.5)	0.052 (0.035, 0.070)	12.0(10.4, 13.1)
CKD	23	9	14	699	11.0 (7.5, 16.0)	0.020 (0.013, 0.035)	11.6(10.7, 13.5)
Neoplasia	19	1	18	745	9.1 (5.9, 13.8)	0.024 (0.013, 0.035)	11.7(10.8, 13.1)
Hyperthyroidism	18	6	12	713	8.6 (5.5, 13.2)	0.017 (0.007, 0.026)	11.1(10.5, 12.1)
Diabetes Mellitus	4	1	3	741	1.9 (0.7, 4.8)	0.004 (0.000, 0.009)	12.2 (12.2, 12.7)

Cats were enrolled at middle age (between 7–10 years).

Prevalence includes all new and existing cases of each disease, whilst incidence rates exclude pre-existing cases.

^1^Number of cats that were diagnosed with each condition over the entire study period.

^2^Number of cats with each condition at time of enrolment (including those cats with pre-existing diagnosis and those diagnosed at the enrolment examination).

^3^Incident cases = Total number of cats diagnosed with each condition minus number of cats with the condition present at enrolment.

^4^Time at risk equates to the total number of cat-years from enrolment date up to either diagnosis of disease, death, withdrawal from study or the end of the study period.

^5^Prevalence reported as mean percentage, with the 95% confidence interval in brackets.

^6^Incidence rate reported as mean percentage per cat year, with the 95% confidence interval in brackets.

^7^Median age at diagnosis (in years), with inter-quartile range in brackets, which excludes both pre-existing cases and also those diagnosed at initial enrolment.

CKD, chronic kidney disease; OE, orthopedic examination.

**Figure 2:**
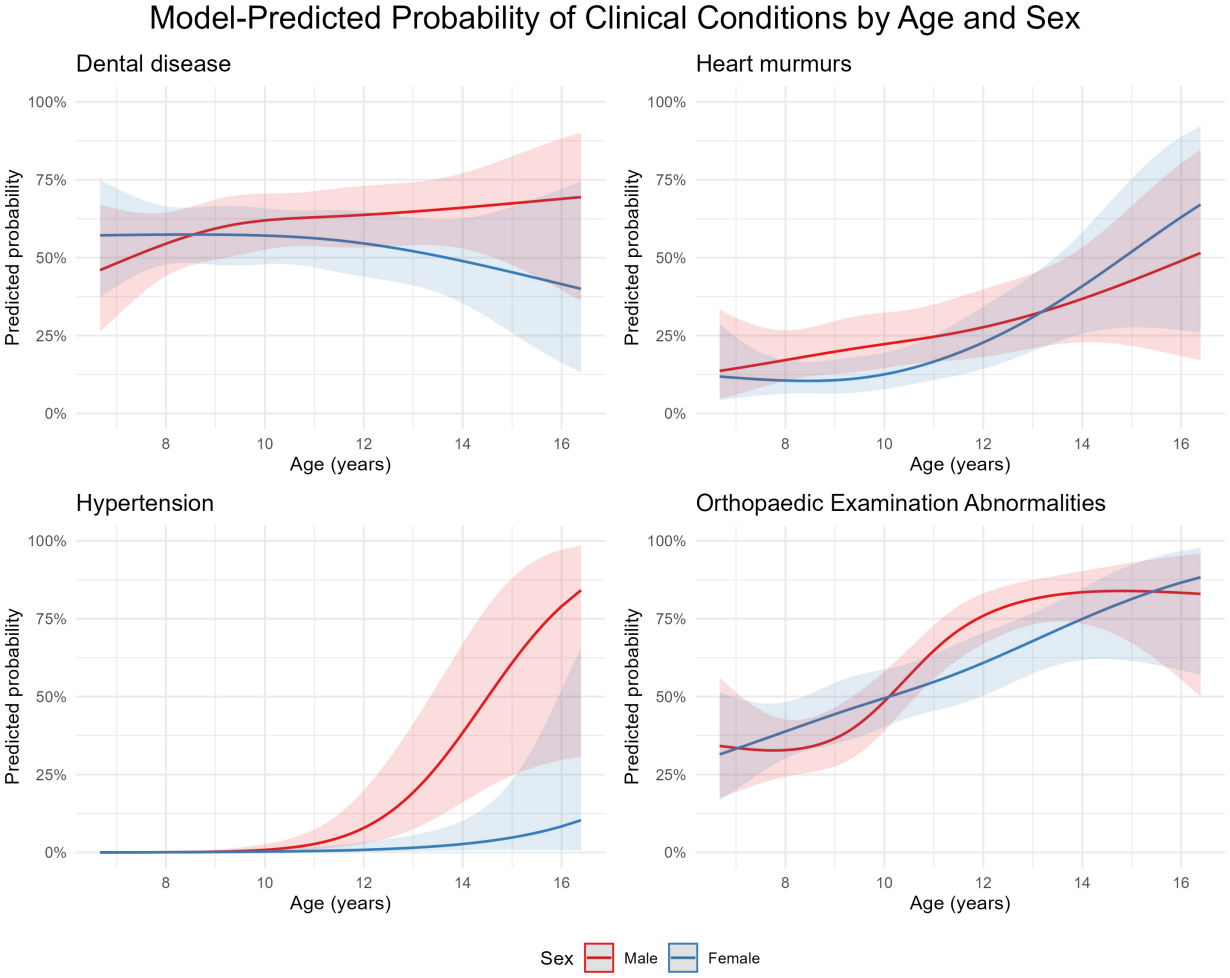
Results of binomial mixed-effects models investigating the predicted probabilities of dental disease, heart murmurs, hypertension and orthopedic examination abnormalities in 209 study cats. Each condition was modeled with the fixed effects of age (modeled using natural cubic splines to three degrees of freedom to allow for non-linear changes with age), sex and the interactions of age and sex, with individual cat subjects as random effect to account for repeated measures within cat subjects.

The results of the binomial mixed-effects models investigating predicted probabilities of dental disease, hypertension, heart murmurs and OE abnormalities by age and sex are presented in Figure 2, with full model outputs provided in the supplementary information (Supplementary Table 4). Age (modeled using natural cubic splines to three degrees of freedom) was significantly associated with hypertension (LRT: χ^2^_6_ = 161.2, *P* < 0.001), heart murmurs (LRT: χ^2^_6_ = 28.8, *P* < 0.001) and OE abnormalities (LRT: χ^2^_6_ = 81.2, *P* < 0.001), but not dental disease (LRT: χ^2^_6_ = 4.3, *P* = 0.630). Sex was significantly associated with hypertension (LRT: χ^2^_4_ = 10.8, *P* = 0.028). The predicted probability of hypertension increased markedly in male cats after approximately 10 years of age (Figure 2).

Cumulative event curves showing the age of development for each morbidity within the cohort are shown in the supplementary information (Supplementary Figure 3).

### 3.4 Other morbidities

Several other morbidities were either pre-existing or developed in the cohort during the study. Two cats had feline immunodeficiency virus (FIV), and both were euthanased, one cat due to the FIV diagnosis and the other due to concurrent stomatitis and an oral mass. Nine cats had upper respiratory tract infection, based on signs of sneezing or conjunctivitis at one or more visits. A further four cats had chronic gastrointestinal disease, whilst one cat had cerebellar hypoplasia and concurrent chronic rhinitis. Liver disease and pancreatitis were present in three and one cats, respectively. Idiopathic hypercalcaemia, non-specific dermatitis, otitis externa and cognitive dysfunction syndrome were recorded in one, four, three and one cats, respectively.

### 3.5 Multimorbidity in study cats

Associations with the following morbidities and abnormalities on examination were assessed: diabetes mellitus, hyperthyroidism, neoplasia, CKD, hypertension, heart murmurs (of any grade), OE abnormalities (of any grade) and dental disease. Of the 209 cats, only nine cats (4%) did not develop any of these conditions, whilst 25 cats (12%) developed one morbidity, 44 cats (21%) developed two morbidities, 78 cats (37%) developed three morbidities, 40 cats (19%) developed four morbidities, eight cats (4%) developed five morbidities, and five cats (2%) developed six morbidities. The most common grouping of morbidities included dental disease, OE abnormalities and heart murmurs, which occurred in 103 (49%) of the cats, with or without additional morbidities. The different combinations of morbidities seen are shown in the supplementary information (Supplementary Table 5), whilst pairings of different conditions are summarized in Table 2. In the 41 cats that were diagnosed with hypertension, 28 (68%) also had a detectable heart murmur on at least one examination, nine (22%) had concurrent hyperthyroidism, and a further nine (22%) had concurrent CKD. None of the cats that developed hypertension, hyperthyroidism, diabetes mellitus or CKD over the course of the study had these conditions in isolation.

**Table 2:** Pairwise co-occurrence of chronic conditions in study cats.

Condition	Heart murmur	Hypertension	Hyperthyroidism	Diabetes mellitus	CKD	Neoplasia	Dental disease	OE abnormalities	No other morbidities
Heart murmur (*n* = 124)		28 (23)	15 (12)	3 (2)	13 (11)	11 (9)	110 (89)	113 (91)	3 (2)
Hypertension (*n* = 41)	28 (68)		9 (22)	1 (2)	9 (22)	4 (10)	39 (95)	37 (90)	0 (0)
Hyperthyroidism (*n* = 18)	15 (83)	9 (50)		0 (0)	4 (22)	1 (6)	17 (94)	16 (89)	0 (0)
Diabetes mellitus (*n* = 4)	3 (75)	1 (25)	0 (0)		1 (25)	0 (0)	4 (100)	3 (75)	0 (0)
CKD (*n* = 23)	13 (57)	9 (39)	4 (17)	1 (4)		3 (13)	23 (100)	22 (96)	0 (0)
Neoplasia (*n* = 19)	11 (58)	4 (21)	1 (5)	0 (0)	3 (16)		17 (90)	17 (90)	1 (5)
Dental disease (*n* = 176)	110 (63)	39 (22)	17 (10)	4 (2)	23 (13)	17 (10)		153 (87)	13 (7)
OE abnormalities (*n* = 172)	113 (66)	37 (22)	16 (9)	3 (2)	22 (13)	17 (10)	153 (89)		8 (5)

Values represent the number (and percentage) of cats with the row condition that also had the column condition.

Percentages are calculated using the total number of cats with the row condition as the denominator.

“No other morbidities” indicates cats diagnosed with the row condition in the absence of any other recorded morbidity.

For example, 124 cats had a heart murmur, of which 28 (23%) also had hypertension and three (2%) had no co-occurring morbidities.

OE, orthopedic examination; CKD, chronic kidney disease.

**Figure 3:**
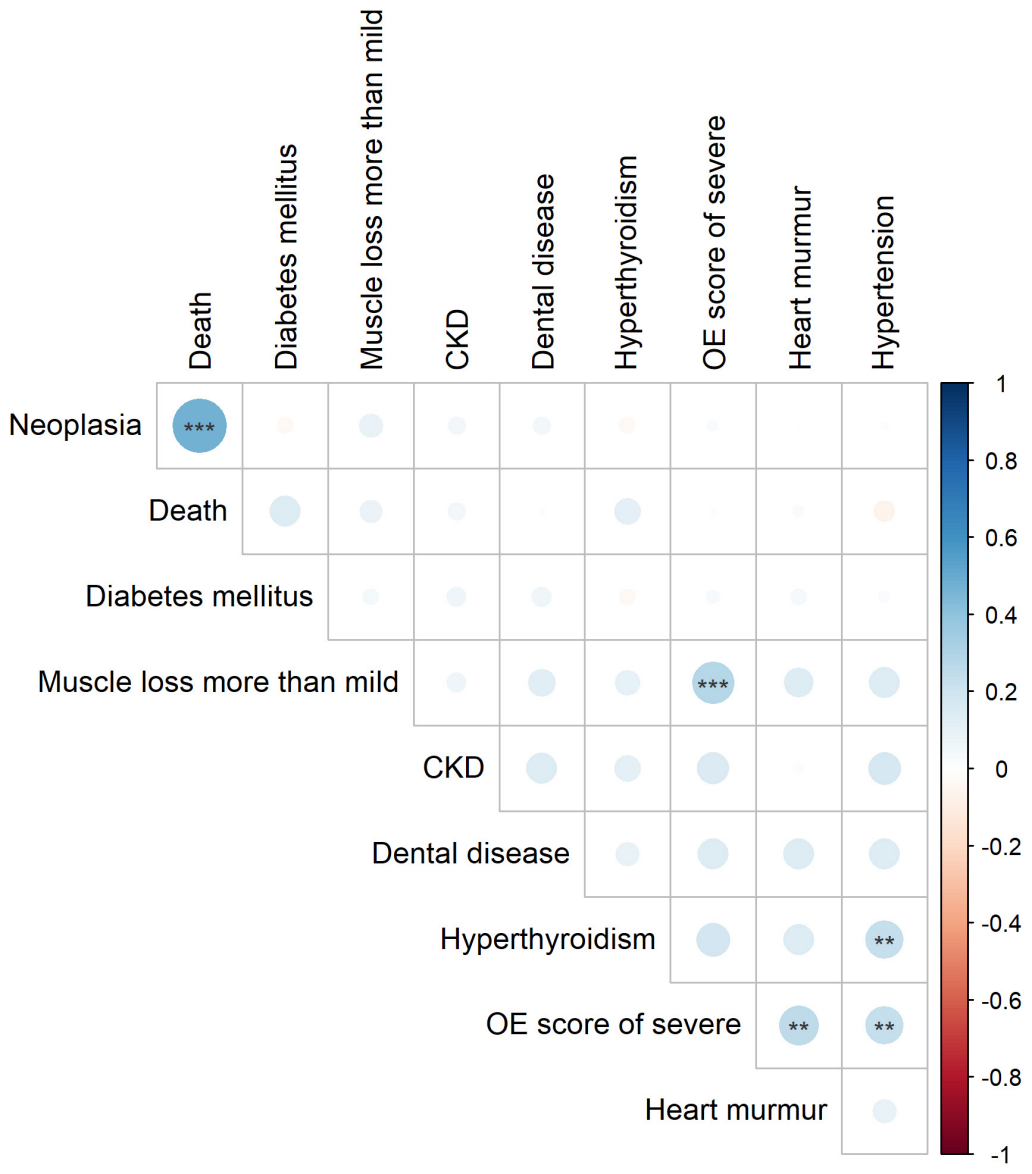
Correlation matrix examining associations of different comorbidities and mortality diagnosed in the 209 study cats. Color of circles in the heat map indicate Phi coefficient from −1 (red) to +1 (blue). The areas of circles show the absolute value of corresponding correlation coefficients. Stars denote FDR-adjusted *P*-value significance (*P < 0.05, **P < 0.01, ***P < 0.001). OE score of severe: an orthopedic examination score of ≥5/14 during the study. Muscle loss more than mild (combined muscle condition score from 10 skeletal landmarks) < 20/30, with each landmark graded from 0 (severe muscle loss) to 3 (no muscle loss). CKD: chronic kidney disease; OE: orthopedic examination.

Correlation analysis revealed certain pairings of morbidities that developed in the cats over the study period (Figure 3). Hypertension was positively associated both with hyperthyroidism (ϕ = 0.23, FDR-adjusted *P* = 0.007) and an OE score of ≥5/14 (ϕ = 0.23, FDR-adjusted *P* = 0.007). Having an OE score of ≥5/14 was also associated with moderate to severe muscle loss (ϕ = 0.28, FDR-adjusted *P* < 0.001) and the presence of a heart murmur (ϕ = 0.25, FDR-adjusted *P* = 0.004). Finally, death during the study was positively associated with a diagnosis of neoplasia (ϕ = 0.47, FDR-adjusted *P* < 0.001).

**Figure 4:**
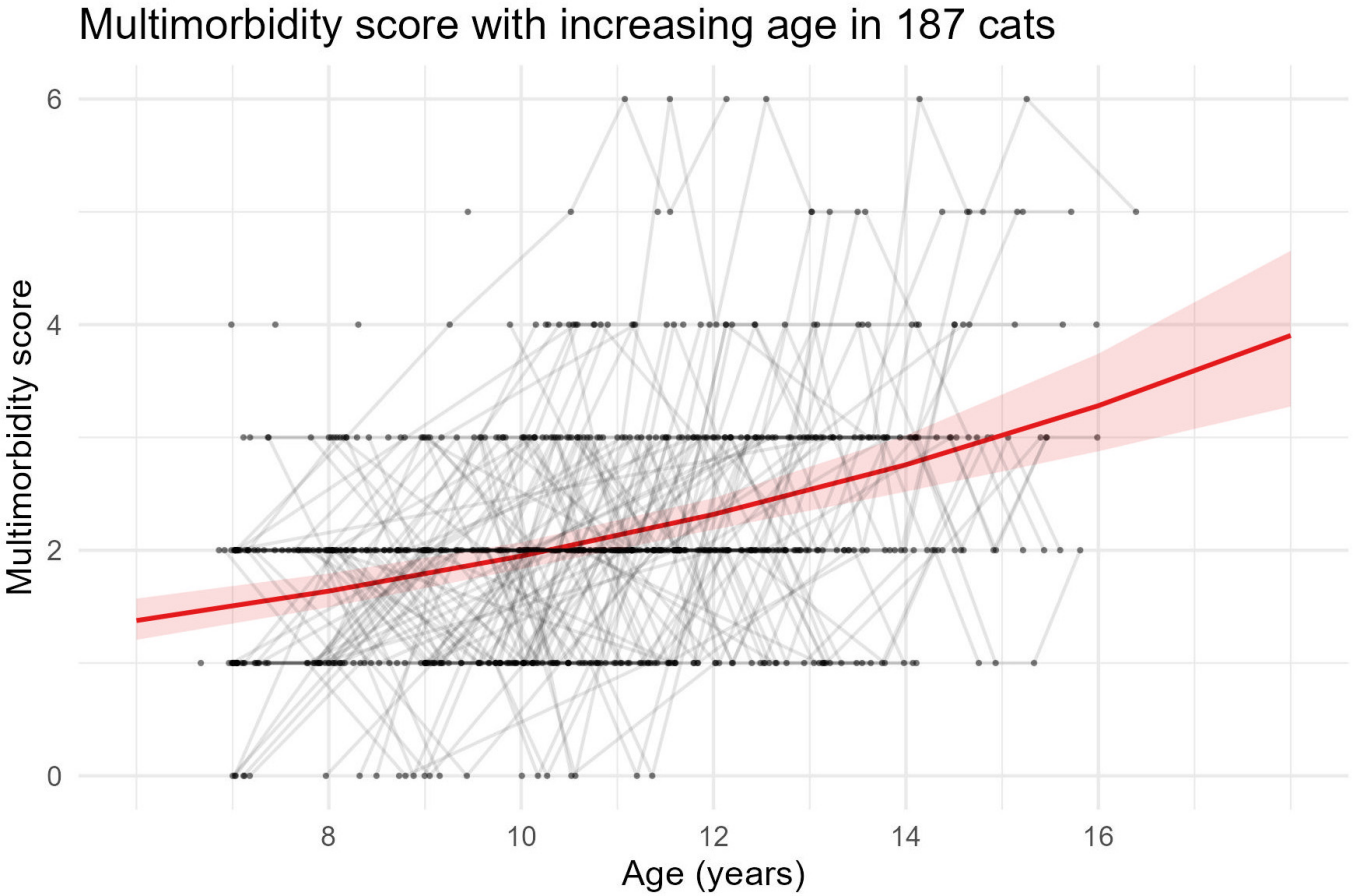
Multimorbidity scores with increasing age from 723 veterinary examinations of 187 study cats. Individual points depict the multimorbidity scores (the cumulative number of chronic conditions or examination abnormalities recorded at each visit) in each cat, with conditions including: chronic kidney disease, neoplasia, hypertension, hyperthyroidism, heart murmur, diabetes mellitus, dental disease and orthopedic examination abnormalities, with a total score of zero to eight. Gray lines connecting points represent repeated measures within cat subjects. The solid red line represents the fit from a Poisson generalized mixed-effects model examining changes in comorbidity score with age, whilst the shaded red ribbon represents the 95% confidence interval.

A multimorbidity score could be calculated for 723 veterinary examinations from 187 cats, where complete diagnostic information was available. Age was associated with an increase in multimorbidity score (incidence rate ratio 1.09, 95% CI 1.07, 1.12; *P* < 0.001; Figure 4). Of these 723 examinations, no cat >12 years had a multimorbidity score of 0 (Figure 4).

### 3.6 Mortality in study cats

Fifty-nine of the 209 cats (28%) died during the study period, with the mean age at death being 11.6 (SD 2.20) years. Forty-eight cats were euthanised, eight cats died without euthanasia, and three cats had no record of whether they died by euthanasia or not. During follow-up, 13/29 (45%) pedigree cats and 46/180 (26%) non-pedigree (domestic short, medium or long-haired) cats died. Causes of death, classified by disease grouping, are shown in Table 3. The five most common causes of death were neoplasia (18/59 cats, 31%), cardiovascular (8/59 cats, 14%), endocrine (5/59 cats, 9%), renal (4/59 cats, 7%) and sudden unexplained death (4/59 cats, 7%). Of the 18 cats that died from neoplasia, six were attributed to lymphoma, three had a gastrointestinal tumor, two had an oral neoplasm and two had an unspecified abdominal mass. There was one case each of renal carcinoma, pancreatic mass, cardiac neoplasm, liver neoplasm, and a malignant neoplasia of unknown type. Cardiovascular conditions leading to death included heart failure (five cats) aortic thromboembolic disease (ATE, two cats) and both heart failure and ATE (one cat). The cats that had cause of death classed as endocrine disorders included two cats with diabetes mellitus, and three cats with hyperthyroidism. Three other cats had both heart failure and concurrent hyperthyroidism, recorded separately from those with either cardiovascular disease or hyperthyroidism (Table 3). Finally, the four cats with a renal disorder as cause of death included three with CKD, and one with acute kidney injury.

**Table 3:** Causes of death in the 59 cats that died during the study period.

Cause of death	Number of cats	Percentage (%)
Neoplasia	18	31
Cardiovascular disease	8	14
Endocrine disorder	5	9
Renal disorder	4	7
Sudden unexplained death	4	7
Cardiovascular disease and endocrine disorder	3	5
Poor quality of life	3	5
Gastrointestinal disorder	2	3
Multiple morbidities	2	3
Neurological	2	3
Unknown	2	3
Anemia	1	2
Hepatopathy	1	2
Infectious disease	1	2
Pancreatic disorder	1	2
Trauma	1	2
Urinary disorder	1	2
Total	59	100

#### 3.6.1 Life expectancy of study cats

**Figure 5:**
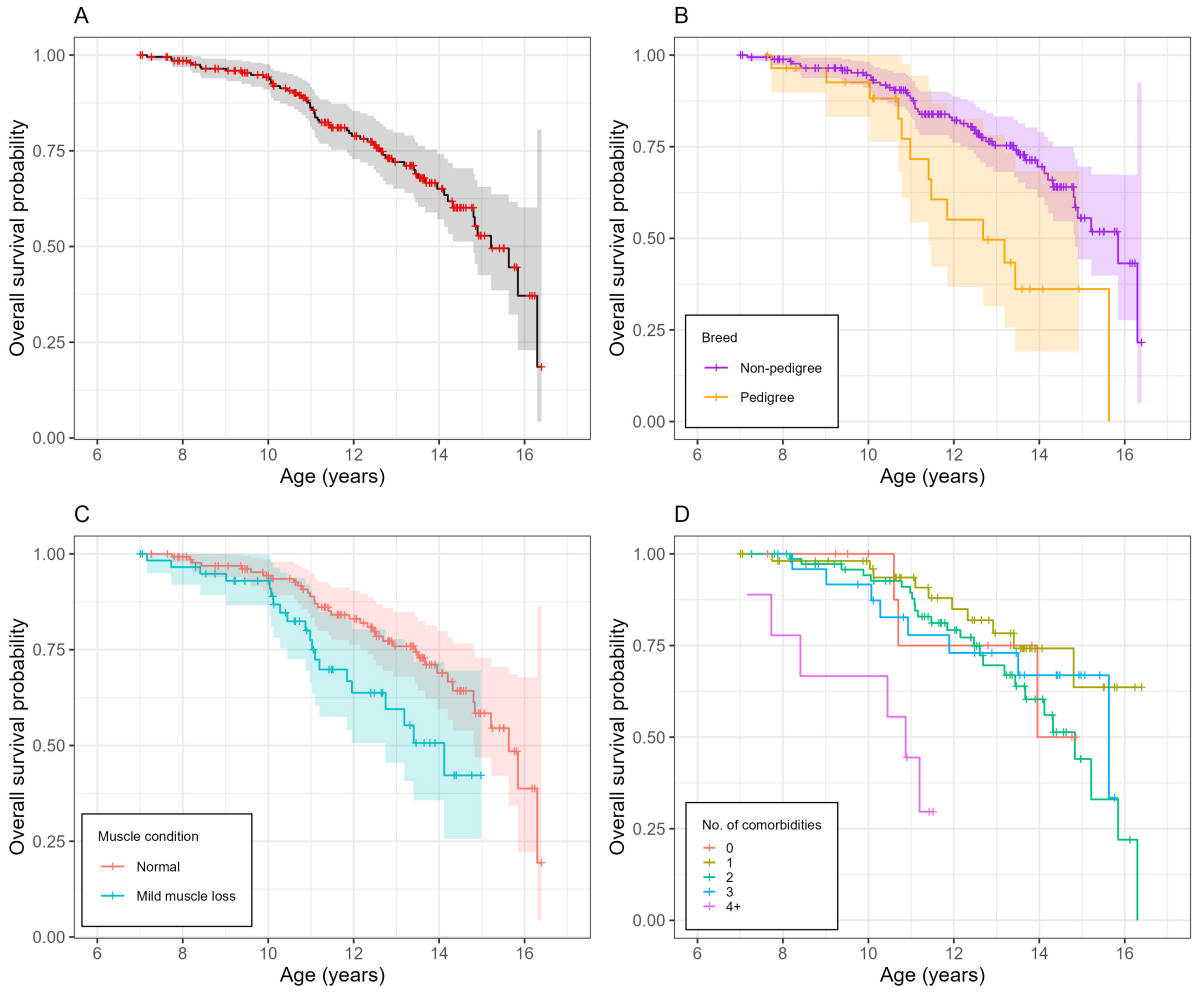
Kaplan-Meier curve showing survival probability for **(A)** all 209 study cats; **(B)** 209 study cats stratified by breed; **(C)** 198 cats stratified by muscle condition at enrolment; **(D)** 178 study cats stratified by number of morbidities at enrolment. The solid lines and shaded ribbon represent survival probability and 95% confidence interval, respectively (not shown in Figure **(D)** due to the number of survival curves), whilst crosses denote censored data (i.e. age at which cats either dropped out of study or their age at the end of the study period if still alive). Median survival time (i.e. median life expectancy) was 5,557 days (15.2 years).

Median survival time, equivalent to the median life expectancy, was 15.2 year (5,557 days), with the lower limit of the 95%-CI being 14.8 year (5,407 days; Figure 5A). It was not possible to estimate the upper 95%-CI due to insufficient deaths during the study period. The overall probability of a study cat being alive at 15 year was 53% (95%-CI: 43%, 66%). The median life expectancy of pedigree cats was 12.7 year (4,633 days) compared with 15.8 year (5,787 days) in non-pedigree cats (95%-CI again not reported due to insufficient numbers of events) (Figure 5B).

#### 3.6.2 Factors associated with mortality

Survival analyses were performed on a complete-case dataset including 190 cats with all included predictor variables recorded at enrolment (apart from the univariable Cox proportional hazards model investigating multimorbidity score and mortality, where only 178 cats had a multimorbidity score assigned at enrolment). During the study period, 54 of the 190 cats died and 55 were lost to follow up. The median follow up time was 4.3 years (IQR 1.1, 5.7 years, range 0.0, 6.9 years).

##### 3.6.2.1 Univariable Cox proportional hazards analyses

The results of the univariable Cox proportional hazards analyses investigating the association between clinical factors at enrolment and the hazard of mortality are presented in Table 4.

**Table 4:** Results of univariable Cox proportional hazards models investigating the association of separate predictor variables at enrolment to the study and mortality in 190 cats.

Characteristic	*N*	HR	95%-CI	*P*
Body condition score				
5	53	—	—	—
3–4	31	2.48	1.08, 5.67	**0.031**
6	48	1.55	0.67, 3.60	0.308
7–9	58	1.99	0.91, 4.35	0.086
Muscle condition score				
No muscle loss	132	—	—	—
Mild muscle loss	58	1.95	1.09, 3.48	**0.024**
Bodyweight (kg)				
	190	0.99	0.76, 1.28	0.928
Sex				
Female	99	—	—	—
Male	91	0.99	0.58, 1.68	0.959
Breed				
Non-pedigree	162	—	—	—
Pedigree	28	2.54	1.35, 4.76	**0.004**
Chronic kidney disease				
No	180	—	—	—
Yes	10	1.66	0.60, 4.62	0.332
Hypertension				
No	180	—	—	—
Yes	10	1.13	0.41, 3.17	0.809
Hyperthyroidism				
No	184	—	—	—
Yes	6	3.1	0.95, 10.1	0.062
Dental disease				
No	74	—	—	—
Yes	116	1.49	0.83, 2.68	0.179
Heart murmur grade				
None	132	—	—	—
I-II/VI	47	1.5	0.81, 2.78	0.193
≥III/VI	11	2.26	0.88, 5.81	0.091

Each predictor variable was modeled separately.

Models were based on 190 cats and 54 deaths.

Cats were aged 7–10 years at enrolment and followed for a median of 4.3 years (IQR 1.1 – 5.7 years).

Age was used as the underlying time scale to inherently account for age in the models.

The proportional hazards assumption was violated for body condition score and bodyweight, therefore these results should be interpreted with caution.

HR, hazard ratio; CI, confidence intervals.

*P*-values shown in bold denote those that met the threshold for significance (*P* < 0.05).

Sex was not associated with hazard of mortality (HR 0.99; 95% CI 0.58, 1.68; *P* = 0.959). Pedigree cats had a significantly greater hazard of death compared with non-pedigree cats (HR 2.54; 95% CI 1.35, 4.76; *P* = 0.004) (Figure 5B).

None of the cats had muscle loss graded as moderate or severe at enrolment; however, cats with mild muscle loss had nearly twice the hazard of death compared with cats with no muscle loss (HR 1.95; 95% CI 1.09, 3.48; *P* = 0.024) (Figure 5C).

Cats with a BCS of 3–4/9 had an increased hazard of death compared with those with a BCS of 5/9 (HR 2.48; 95% CI 1.08, 5.67; *P* = 0.031). However, the proportional hazards assumption was violated for this analysis, indicating that the effect of BCS on hazard was not constant over time and, therefore, should be interpreted with caution (Supplementary Figure 4).

In the 178 cats where an enrolment multimorbidity score could be assigned, hazard of death during the study was six times greater in those with ≥4 comorbidities (HR 6.30, 95% CI 1.5, 26.1; *P* = 0.011; Figure 5D). Proportional hazards assumptions were met for this model (z-test global *P* = 0.577).

##### 3.6.2.2 Time-dependent Cox proportional hazards model examining changes in baseline hazard with age for enrolment body condition score categories

The results of the univariable Cox proportional hazards model investigating the association of BCS categories at enrolment and survival violated the proportional hazards assumption, suggesting a change in baseline hazard depending on the age of the cat. Therefore, a time-dependent Cox model was also fit, separating survival at ages < 11 years and ≥11 years, based on examining Schoenfeld residual plots.

BCS group was not associated with survival in cats < 11 year, although BCS 3–4 was borderline significant (*P* = 0.055). However, in cats ≥11 year, those with BCS 7–9 at enrolment had an increased hazard of death compared to cats with BCS 5 at enrolment (HR 5.04, 95% CI 1.62, 15.69, *P*= 0.008; Table 5). The proportional hazards assumption was satisfied after splitting survival time in this manner (z-test global *P* = 0.40).

**Table 5:** Time-dependent Cox model investigating the association between body condition score (BCS) categories at enrolment and survival in study cats with survival split at 11 years.

BCS group	Baseline *N*	< 11 years			≥11 years		
		HR	95% CI	*P*	HR	95% CI	*P*
5 (ref)	53	—	—	—	—	—	—
3–4	31	2.75	0.98–7.71	0.055	1.98	0.79–7.98	0.713
6	48	0.74	0.21–2.62	0.638	2.94	0.88–9.85	0.121
7–9	58	0.45	0.11–1.81	0.263	5.04	1.62–15.69	**0.008**

HR, hazard ratio; CI, confidence interval.

The time-dependent Cox model stratifies survival at 11 years of age, creating 311 cat-intervals; cats surviving past 11 years contribute to both < 11-year and ≥11-year strata.

Survival time was split at 11 years of age; HRs are shown separately for < 11 years and ≥11 years.

The model was based on 190 cats and 54 deaths.

Baseline N represents cohort size at enrolment. BCS 5/9 was the reference category.

*P*-values shown in bold met the threshold for significance (*P* < 0.05).

##### 3.6.2.3 Multivariable Cox proportional hazards regression model

The five top-performing models and their BIC are provided in the supplementary information (Supplementary Table 6). The best-fit model (LRT *P* = 0.002) had modest discriminatory ability (C-index = 0.611) and contained breed and MCS grade at enrolment as the main predictor variables (Table 6). Age (in decimal years, calculated as days divided by 365.25 to account for leap years) was used as the underlying time scale, thereby inherently adjusting for age and reducing potential confounding by age. In the final model (54 deaths in 190 cats), mortality was positively associated with breed group and pedigree cats had 2.62 times greater hazard of death over the study compared with non-pedigree cats (HR 2.62, 95% CI 1.39, 4.93; *P* = 0.003; Table 6). Cats with mild muscle loss at enrolment had twice the hazard of death compared with cats with no muscle loss (HR 2.02, 95%-CI 1.13, 3.60, *P* = 0.018; Table 6).

**Table 6:** Multivariable Cox proportional hazards model examining risk factors at enrolment for mortality in 190 study cats.

Characteristic	Number	Hazard ratio	95%-CI	*P*-value
Breed			
Non-pedigree	162	—	—	—
Pedigree	28	2.62	1.39, 4.93	**0.003**
Muscle condition			
Normal	132	—	—	—
Mild muscle loss	58	2.01	1.13, 3.60	**0.018**

95%-CI, 95% confidence interval.

Muscle condition was scored using combined scores from 10 skeletal landmarks each graded from 3 (no muscle wastage) to 0 (severe muscle wastage): mild muscle loss 20–29/30; normal 30/30 (reference category). Age was used as the underlying time scale to inherently account for age in the models. *P*-values shown in bold met the threshold for significance (*P* < 0.05).

Internal validation demonstrated modest discrimination (optimism-corrected Dxy = 0.214, corresponding to a C-index of approximately 0.61). The model showed minimal optimism (0.008) and good calibration (slope = 0.995), indicating low risk of overfitting.

## 4 Discussion

In this observational cohort study, the most prevalent disease identified was dental disease, which affected 84% of cats. This prevalence is greater than that reported in previous studies, with estimates of 15% in UK cats (16) and 53% in cats >5 years of age from USA veterinary hospitals (49). The prevalence of dental disease was probably greater in this study due to the older average age of the cats studied, its longitudinal nature and that a careful oral cavity examination was undertaken at each health check, with any dental problems recorded. Overall prevalence of dental disease did not increase with age in this study, which was similar to findings from a recent study of ~18,000 cats attending primary care practice in the UK, where the risk of periodontal disease plateaued after 12 years age (16). The reason for this is not clear but might be because study cats were often treated for their dental disease after it had been identified. The incidence rate for dental disease was estimated 0.398 cases per cat-year, but this might have been an under-estimate due to left censoring; in this respect, dental disease was already present at the time of enrolment in over half the study cats (129/209). A more accurate incidence estimate could be obtained from future studies using cat cohorts recruited earlier in life (50).

Orthopedic examination abnormalities were identified in 82% of the study cats, with an incidence rate of 0.375 new cases per cat-year which included any level of severity of OE abnormalities. The prevalence of radiographic osteoarthritis in the appendicular joints in cats was 61% in one joint and 48% in two or more joints in a study of 100 cats, with prevalence increasing with age (51). However, false positives are possible on orthopedic examination in cats, not least when it is used as the sole method of assessment, for example, when investigating possible pain (32). Conversely, radiographic signs of osteoarthritis in cats do not always correspond with clinical signs of pain or impaired mobility (52). Diagnosing clinical musculoskeletal impairment in aging cats can, therefore, prove somewhat challenging (53). Cat-friendly techniques to reduce stress during veterinary examinations can improve the confidence of pain assessments (54). The use of clinical metrology instruments such as the Feline Musculoskeletal Pain Index can further aid the diagnosis of musculoskeletal disorders in cats, and be useful in monitoring the response to treatment (55).

An OE score of ≥5 was also associated with hypertension, an audible heart murmur on cardiac auscultation and moderate-to-severe muscle loss. Muscle wastage often occurs secondary to chronic pain in cats with degenerative joint disease (56, 57), and can also occur in old age in the absence of other diseases (termed sarcopenia). In humans, sarcopenia leads to impaired mobility and frailty (58), but decreases in muscle strength or performance have not yet been investigated in aging cats. Osteoarthritis has been linked to an increased risk of cardiovascular disease (59, 60), although the underlying reason for this is complex and still not fully understood. Hypertension is also associated with an increased odds of having osteoarthritis in people, which may not be due solely to chronic pain, but also due to the role the vascular system is thought to play in joint disease (61, 62). The outcomes of orthopedic assessments in the CatPAWS cohort will be investigated in a future study which will include how the severity of OE abnormality changed with age in the cats.

Heart murmurs were identified in 59% of cats in this study, which is similar to previous studies where a prevalence of 60% was reported in cats >9 years of age (27). The prevalence of heart murmurs increased with age but was not associated with the sex of the cats. The incidence rate of newly detected heart murmurs was 0.155 cases per cat-year. Unfortunately, it was not possible to confirm whether the heart murmurs identified were associated with underlying structural cardiac abnormalities because echocardiography was not performed. In a study of 856 cats with audible heart murmurs referred for echocardiography, over half did not subsequently have echocardiographic evidence of heart disease (28). The presence of a heart murmur also had low predictive power for identifying hypertrophic cardiomyopathy (HCM) in a study of 780 cats that underwent echocardiography (27). However, in that study, the presence of a heart murmur (especially grade III/VI and greater), increasing age, male cats and overweight cats were risk factors for HCM in a multivariable model (27).

Hypertension was identified in 20% of the cohort during the study period, and the prevalence increased as age of the cats increased. This prevalence estimate is marginally greater than the 15% prevalence recently reported in a similar cohort of 185 cats aged >10 years (63), but less than the 40% prevalence reported in a large multicentre study of SBP in 8,884 cats >7 years old (64). The estimated incidence rate of new cases of hypertension in the current study was 0.052 per cat-year; unfortunately, it is not possible to make comparisons with previous studies because, to the authors' knowledge, incidence rates have not previously been documented. Hypertension was also associated with cats having muscle loss and hyperthyroidism; hyperthyroidism being known to cause secondary hypertension (25). Further, there was an increased prevalence of hypertension in male cats >10 years old. More detailed longitudinal modeling to assess further associations of various clinical variables with systolic blood pressure measurements and risk of hypertension is currently being undertaken. This future work which will account for possible confounding factors in this apparent sex difference such as body composition and comorbidities.

The estimated incidence rate of CKD diagnosis was low, at 0.020 cases per cat-year, although overall prevalence was 11%, which is greater than in one recent study of electronic medical records where prevalence was 4% in cats >9 year (65), but less that in a second study where prevalence of CKD (with concurrent azotaemia) was 18% (18). Further, a 50% prevalence of CKD was estimated in a study of 86 randomly selected cats aged 6 months to 20 years, whilst the prevalence in 128 cats with degenerative joint disease was 68% (66). The discrepancies amongst studies are likely due to differences in the populations investigated, the study design and the diagnostic procedures. In this respect, the current study used proactive screening veterinary examinations of cats, meaning that cats with early CKD might have been identified earlier than expected, and before the onset of clinical signs. Further, although similar methodology to that of Finch et al. (18) was used to diagnose CKD, the approach used by Marino et al. (66) was different. In that study the abdominal radiographs were taken in all cats to determine kidney size and better identify cases of non-azotaemic CKD (*e.g.*, IRIS stage I), which probably explains the greater prevalences recorded. The approach to CKD diagnosis used in our study may also have underestimated CKD prevalence and incidence by overlooking IRIS stage I and early IRIS stage II CKD. We also recognize that using a threshold of serum creatinine in the current study of >177 μmol/L for laboratory analysers where the upper reference interval was 212 μmol/L could risk overestimating CKD diagnosis; however, it is also noted that the IRIS guidelines do not consider laboratory differences.

Multimorbidity was common in the aging cats. Only 9/209 cats remained free of any abnormality on clinical examination over the study, and 175/209 (84%) developed two or more abnormalities. The most common groupings of conditions were dental disease, OE abnormalities, and heart murmurs (with or without additional morbidities), which affected 103/209 cats (49%). All cats with either hypertension, hyperthyroidism, diabetes mellitus or CKD had developed other comorbidities by the end of the study. However, this analysis did not take into consideration grades of heart murmur or whether the heart murmur was indicative of clinical signs of heart disease. Likewise, any abnormality on OE was included without accounting for the severity of OE abnormalities and whether the cat had other metrics of musculoskeletal disorders.

Multimorbidity with aging is reported in many species including dogs (67) and humans (68), and poses a challenge for the care of aging patients. The need for polypharmacy to treat different conditions leads to an increase in potential for drug-interactions (69), and trying to manage multiple conditions simultaneously whilst prioritizing overall quality of life can be difficult to balance. In cats, the dilemma of managing cats with both hyperthyroidism and CKD, with or without common musculoskeletal disorders such as osteoarthritis, has been well-documented (12, 13). Hyperthyroidism can mask CKD by various mechanisms, including the increase in glomerular filtration rate caused by thyroxine excess, and treating hyperthyroidism can then lead to an increase in biomarkers such as creatinine associated with CKD (13). Given that common treatments for musculoskeletal pain, such as non-steroidal anti-inflammatory drugs (NSAIDs), can be associated with renal toxicity, there are concerns about their use in cats with CKD (12). However, not providing analgesia to cats with a painful musculoskeletal condition can impact welfare and the safety of long-term NSAID therapy in cats with CKD remains unknown (12).

The process of aging is a risk factor for the development of morbidities, and therapies (both pharmaceutical and non-pharmaceutical) targeting the aging process aim to be a broad-spectrum preventative measure for multiple diseases of aging (70). In experimental studies in mice, drugs such as rapamycin and metformin have been found to prolong healthspan and lifespan (71–75). In dogs, the effect of rapamycin treatment on lifespan and healthspan is currently being investigated (76). Rapamycin has been approved by the FDA in the US for the treatment of subclinical HCM in cats, and reduces maximal thickness of the left ventricle compared to placebo (77). The effect of rapamycin treatment for CKD in cats is currently also under investigation (78). No studies have yet reported whether rapamycin treatment results in an overall increased healthspan or lifespan in cats.

Nutrition also plays a role in healthy aging. Interventions such as caloric restriction (CR) have been known for many decades to impact longevity in short-lived animal models such as mice (Fontana et al., 2010). The long-term impacts of CR have not been fully explored, and longevity outcomes may depend on the age of the subject (79). Older aged rats exposed to CR, for example, had worsening cognitive function and frailty levels (Prvulovic et al., 2022). The effects of dietary supplements and nutraceuticals, such as omega-3 fatty acids and antioxidants including vitamin E have been studied in *C. elegans*, fruit flies and laboratory mice with mixed results (80). Increased intake of oily fish containing omega-3 fatty acids and antioxidants have been found to decrease biological age as measured by epigenetic-clock based biomarkers in humans (81). In cats, 30 cats aged 7 years and above fed a diet supplemented with vitamin E, β-carotene, chicory root (source of prebiotic) and *n*-3 and *n*-6 fatty acids over 5 years lived significantly longer and has less disease incidence than those receiving a control diet (82).

The mortality rate in this study was 28% (59/209 cats), although this might be underestimated given the fact that many (59/209) cats were withdrawn prematurely and some of these might also have died before the end of the study period. The median life expectancy was 15.2 year, which is similar to some previous reports (14.0 years (3); 15.8 years (83)) but greater than others (11.8 years (84); 11.7 years (85)). Although the present study benefits from its prospective longitudinal cohort design, the sample size was smaller than in the four previous studies that analyzed data from thousands of cats using electronic health records. Furthermore, cats were also only followed from middle age, which might lead to an overestimate of life expectancy since cats dying at a younger age would automatically be excluded. The top causes of death in this current cohort were neoplasia (18 cats, 31% of deaths) and cardiovascular disease (eight cats, 14% of deaths), followed by endocrine disorders (including hyperthyroidism and diabetes mellitus; five cats, 9%), renal disorders (four cats, 7%) and sudden death (four cats, 7%). This contrasts with the O'Neill et al. (3), study where renal disorders were the top cause of death, estimated at 13.6%, with the next most common causes being non-specific illness (12.6%) and neoplasia (12.3%), and cardiac disease being the cause in 4.6% of deaths. However, in that study, mass lesions were included as a separate category (3), which might have resulted in deaths attributed to the neoplasia category being under-estimated. Recently, another prospective cohort study of aging in cats (the “Bristol Cats” study) reported a mortality rate of 362/2,444 cats before the age of 9 years, with the most common cause of death (46% of deaths) being road traffic accidents (22). None of the cats in the present study died from a road traffic accident; it is likely that the risk of road traffic accidents decreases in older cats as they may spend less time outdoors and be less active, as well as having potentially learnt how to avoid road vehicles. Furthermore, before aged 9 years, 16.3% of pedigree cats died whereas 12.6% mixed-breed cats died, possibly showing that pedigrees have a greater mortality rate across life-stages (22).

Risk factors at enrolment for study mortality included being from a pedigree breed, having four or more concurrent morbidities and muscle loss. Age was used as the underlying time-scale, thereby inherently adjusting for age and limiting its potential confounding effect. The associations between pedigree breed, multimorbidity burden and muscle loss with mortality were therefore independent of chronological age at enrolment and unlikely to be explained by age alone. Although, on initial survival analysis using Cox regression, cats that had a BCS of 3–4 at enrolment had an increased hazard of death compared to those cats with BCS 5, the proportional hazards assumption was violated, indicating that the baseline hazard varied with age. After accounting for age-related changes in hazard, BCS at enrolment was no longer associated with survival in cats < 11 years, although cats in overweight and obese body condition (BCS 7–9) were at greater risk of death after 11 years of age. This finding may reflect the small number of cats with a BCS of 3 or less, which necessitated grouping underconditioned cats with those of BCS 4. Cats with a BCS of 4 may be at lesser risk of death before 11 years of age than cats with a BCS of 3, potentially diluting the observed effect in younger cats. These results emphasize the need for cats in middle age to maintain an optimal body condition [currently considered to be BCS 5–6/9; (83)], to maximize longevity. Previous studies have also found that BCS of < 5/9 or 9/9 (83) or a non-ideal body weight (85) negatively affects longevity in cats. This U-shaped relationship between body condition and mortality risk, where individuals who are either very thin or very fat are at greater risk of mortality, is also well documented in humans (86). Body condition score and MCS were found to change with age in a non-linear fashion in this cohort (24), and changes in these markers of body composition with age may also impact survival. This study aimed to examine predictors of mortality from enrolment data, but another important question for future analysis would be to examine whether changes in BCS and MCS over the aging process also have an impact on mortality risk.

In our final Cox regression model, the most important predictors of mortality were breed and muscle loss at enrolment. In the same cohort of cats, muscle condition decreased over middle age, and decreased at a greater rate after age 10 years and in those cats that developed age-related morbidities (24). Furthermore, although body condition increased slightly during middle age, there was a subsequent decline after 10 years suggesting that the changes in BCS and MCS do not necessarily correlate with each other over a cat's lifespan (24). Muscle wastage is common in aging cats both as a consequence of age-related morbidities and due to aging related mechanisms (87) and contributes to the frailty phenotype in people and dogs (88–90). Severe epaxial muscle wastage at 12 years (range 9–17 years) is also associated with decreased lifespan in various dog breeds (90). In a recent pilot study, muscle loss was associated with increased odds of a veterinarian classifying a cat as frail and frail cats had an increased risk of 6-month mortality (91). The finding that muscle condition at middle age can be a predictor of future mortality underlines the importance of both recording MCS in clinical examinations and implementing preventative therapies to maintain muscle mass in aging cats. This could include providing adequate dietary protein and, where appropriate, gentle strength-building activities, such as interactive play or low-impact exercise tailored to the pet's ability. Future work to assess the importance of muscle wastage with frailty in cats, and how it is associated with mobility in aging cats is required to gain more knowledge of the effect of muscle wastage on quality of life. Further work to identify targets to prevent muscle wastage, and the effect of dietary protein would also be of use.

The finding of a shorter average lifespan in pedigree cats in the current study is, perhaps, not surprising because pedigree cats have long been hypothesized to have shorter lifespans than mixed-breed cats due to “inbreeding depression” causing an increased likelihood for the expression of deleterious genes (92). The “pedigree” category in the current study had a relatively small sample size and included several cat breeds, though numbers were too small in most individual breeds to permit a separate analysis. In previous studies, pedigree cats had a shorter lifespan (3, 85, 93), although life expectancy varied amongst different breeds (3, 85). In a recent study of life expectancy in 7,936 UK cats, cats of the Sphynx cat breed had the shortest life expectancy (6.7 years), whilst Burmese cats had the longest life expectancy at year zero (14.4 years) which, interestingly, was greater than the mixed-breed cats in that study (85). Variety in longevity between breeds is likely due to different pre-dispositions to diseases. However, in studies relying on data from visits to veterinary clinics, the recorded lifespans may also be biased if the likelihood of attending a veterinary clinic is increased based on the value or perceived value of an animal (94). Pedigree cats are also becoming more popular in the UK; in 2024, more pedigrees were obtained in the preceding 12 months than mixed-breed cats for the first time since data collection began (95). Therefore, it is important to be aware of the potential health risks that certain breeds of cat have.

There were several limitations with this study. Firstly, the sample size was relatively small, and, therefore, some diseases were uncommon (*e.g.*, diabetes mellitus) meaning that estimates of incidence might be unreliable. Further, stratifying incidence rates by different age ranges, which would have provided additional detail, was explored but was not feasible due to small sample sizes of certain diseases at different ages. Second, the prevalence estimates by age-year group are descriptive statistics and do not take into consideration repeated measures within the same cat subjects and, therefore, may underestimate uncertainty. To overcome this, multi-level models were applied to those diseases with enough events, although a further limitation is the possible effect of survivor bias, with some cats dying or being lost to follow up over the course of the study. Third, some cats had an unknown date of birth, which had been estimated by the owner at the time of acquisition; this might have led to inaccuracies in some estimates although, in fairness, this is likely to be an issue that could affect many such studies. Fourth, orthopedic conditions, such as degenerative joint disease, were mainly identified by OE, but not confirmed definitively with radiography. Likewise, echocardiography was not performed to determine the cause of the heart murmurs in most cats, although such findings were discussed with the owner enabling them to pursue a diagnosis separately (with their primary care veterinarian) if they wished. Determining definitive diagnoses of disease in first opinion clinical veterinary practice can sometimes be challenging either because of owner financial constraints or because doing so would not change the management or treatment plan. Therefore, it was decided that the presence of heart murmurs and not definitively diagnosed cardiac diseases would be discussed in this study. Fifth, some disorders were not analyzed separately including chronic skin conditions, chronic gastrointestinal disease and chronic cat flu, because these diseases occurred in relatively small numbers. Given that this cohort study is ongoing, it might be possible to examine such cases in more detail in the future. Continued data collection will also allow for other diseases to be studied in the same cohort, such as cognitive dysfunction. Sixth, given that cats were enrolled at 7–10 years, there is a risk of left-censoring of the data for estimates of the median age at diagnosis (for specific diseases). Therefore, these estimates are only valid for cats without that pre-existing diagnosis in middle age. Likewise, estimates of median life expectancy may be over-estimated. More accurate estimates will be possible from cohort studies where cats are enrolled from birth. Furthermore, the cats in this study were from one geographic location (the North-West of England) and, therefore, findings might not be generalisable to cats in other locations. Finally, the effect of clustering within owners and veterinary practices the cats were registered with was not accounted for in the analyses.

## 5 Conclusions and future impact

In this 7-year longitudinal cohort study of aging pet cats, almost all cats (96%) developed at least one abnormality on veterinary examination, and multimorbidity was common, with 84% of cats developing two or more abnormalities during the study period. There are several novel findings including estimates of incidence rates of dental disease and hypertension. Having muscle loss during middle age doubled the hazard of death during subsequent follow up, perhaps suggesting the need for implementing interventions to promote maintenance of an optimal body condition as cats age. Future studies will need to examine in more depth the associations and risk factors associated with specific morbidities and assess potential interventions that could delay or reduce disease burden. Overall, our findings emphasize the importance of considering multi-morbidity in aging cats and suggest that strategies to improve healthy aging should begin before or during middle age.

## Data Availability

The original contributions presented in the study are included in the article/Supplementary material, further inquiries can be directed to the corresponding author.

## References

[1] American Veterinary Medical Association. AVMA 2022 Pet Ownership and Demographic Sourcebook. Schaumburg, IL: American Veterinary Medical Association. (2022).

[2] Cats Protection. Cats and their stats (CATS) report. Report UK. East Sussex: Cats Protection. (2023).

[3] O'Neill DG Church DB Mcgreevy PD Thomson PC Brodbelt DC. Longevity and mortality of cats attending primary care veterinary practices in England. J Feline Med Surg. (2015) 17:125–33. doi: 10.1177/1098612X14536176 24925771 PMC10816413

[4] Bellows J Center S Daristotle L Estrada AH Flickinger EA Horwitz DF . Aging in cats: common physical and functional changes. J Feline Med Surg. (2016) 18:533–50. doi: 10.1177/1098612X16649523 27370392 PMC10816677

[5] Sordo L Breheny C Halls V Cotter A Tørnqvist-Johnsen C Caney SMA . Prevalence of disease and age-related behavioural changes in cats: past and present. Vet Sci. (2020) 7:85. doi: 10.3390/vetsci7030085 32640581 PMC7557453

[6] Salt C Saito EK O'flynn C Allaway D. Stratification of companion animal life stages from electronic medical record diagnosis data. J Gerontol A Biol Sci Med Sci. (2023) 78, 579–86. doi: 10.1093/gerona/glac220 36330848 PMC10061556

[7] Morris J. Mammary tumours in the cat:size matters, so early intervention saves lives. J Feline Med Surg. (2013) 15:391–400. doi: 10.1177/1098612X13483237 23603502 PMC10816587

[8] Dowgray N Pinchbeck G Eyre K Biourge V Comerford E German AJ. Aging in cats: owner observations and clinical finding in 206 mature cats at enrolment to the cat prospective aging and welfare study. Front Vet Sci. (2022) 9:859041. doi: 10.3389/fvets.2022.859041 35445099 PMC9014291

[9] Mortier F Daminet S Marynissen S Smets P Paepe D. Value of repeated health screening in 259 apparently healthy mature adult and senior cats followed for 2 years. J Vet Int Med. (2024) 38:2089–98. doi: 10.1111/jvim.17138 38967102 PMC11256131

[10] López-Otín C Blasco MA Partridge L Serrano M Kroemer G. Hallmarks of aging: An expanding universe. Cell. (2023) 186:243–78. doi: 10.1016/j.cell.2022.11.001 36599349

[11] Harrison C Fortin M Van Den Akker M Mair F Calderon-Larranaga A Boland F . Comorbidity versus multimorbidity: why it matters. J Multimorb Comorb. (2021) 11:2633556521993993. doi: 10.1177/2633556521993993 33718251 PMC7930649

[12] Monteiro B Steagall PVM Lascelles BDX Robertson S Murrell JC Kronen PW . Long-term use of non-steroidal anti-inflammatory drugs in cats with chronic kidney disease: from controversy to optimism. J Small Animal Prac. (2019) 60:459–62. doi: 10.1111/jsap.13012 31081136

[13] Geddes R Aguiar J. Feline Comorbidities: Balancing hyperthyroidism and concurrent chronic kidney disease. J Feline Med Surg. (2022) 24:641–50. doi: 10.1177/1098612X221090390 35481810 PMC11107990

[14] Bellows J Center S Daristotle L Estrada AH Flickinger EA Horwitz DF . Evaluating aging in cats: how to determine what is healthy and what is disease. J Feline Med Surg. (2016) 18:551–70. doi: 10.1177/1098612X16649525 27370393 PMC10816674

[15] O'Neill DG Church DB Mcgreevy PD Thomson PC Brodbelt DC. Prevalence of disorders recorded in cats attending primary-care veterinary practices in England. Vet J. (2014) 202:286–91. doi: 10.1016/j.tvjl.2014.08.004 25178688

[16] O'Neill DG Blenkarn A Brodbelt DC Church DB Freeman A. Periodontal disease in cats under primary veterinary care in the UK: frequency and risk factors. J Feline Med Surg. (2023) 25:1098612x231158154. doi: 10.1177/1098612X231158154 PMC10812011 36912667

[17] Jepson RE Brodbelt D Vallance C Syme HM Elliott J. Evaluation of predictors of the development of azotemia in cats. J Vet Int Med. (2009) 23:806–13. doi: 10.1111/j.1939-1676.2009.0339.x 19566846

[18] Finch NC Syme HM Elliott J. Risk factors for development of chronic kidney disease in cats. J Vet Intern Med. (2016) 30:602–10. doi: 10.1111/jvim.13917 26948860 PMC4864943

[19] Fox PR Keene BW Lamb K Schober KA Chetboul V Luis Fuentes V . International collaborative study to assess cardiovascular risk and evaluate long-term health in cats with preclinical hypertrophic cardiomyopathy and apparently healthy cats: the reveal study. J Vet Intern Med. (2018) 32:930–43. doi: 10.1111/jvim.15285 29660848 PMC5980443

[20] Economu L Stell A O'neill DG Schofield I Stevens K Brodbelt D. Incidence and risk factors for feline lymphoma in UK primary-care practice. J Small Animal Prac. (2021) 62:97–106. doi: 10.1111/jsap.13266 PMC7986087 33325082

[21] Williams JL Roberts C Harley R Gruffydd-Jones TJ Murray JK. Prevalence and risk factors for gingivitis in a cohort of UK companion cats aged up to 6 years. J Small Animal Prac. (2024) 65:605–14. doi: 10.1111/jsap.13737 38736278

[22] Taylor AR Mcdonald J Foreman-Worsley R Hibbert A Blackwell EJ. Mortality and life table analysis in a young cohort of pet cats in the UK. J Feline Med Surg. (2025) 27:1098612X251314689. doi: 10.1177/1098612X251314689 40219622 PMC12033825

[23] Dowgray N. An Epidemiological, Clinical and Biomechanical study into age-related changes in 206 middle aged cats. The CatPAW Study. PhD Thesis, University of Liverpool (2020).

[24] Pye CR Dowgray NJ Eyre K Pinchbeck G Biourge V Moniot D . (2025). Longitudinal changes in bodyweight, body condition, and muscle condition in ageing pet cats: findings from the cat prospective ageing and welfare study. Front Vet Sci. (2025) 12:1654002 doi: 10.3389/fvets.2025.1654002 PMC12415779 40927173

[25] Taylor SS Sparkes AH Briscoe K Carter J Sala SC Jepson RE . ISFM consensus guidelines on the diagnosis and management of hypertension in cats. J Feline Med Surg. (2017) 19:288–303. doi: 10.1177/1098612X17693500 28245741 PMC11119534

[26] Côté E Manning AM Emerson D Laste NJ Malakoff RL Harpster NK. Assessment of the prevalence of heart murmurs in overtly healthy cats. J Am Vet Med Assoc. (2004) 225:384–8. doi: 10.2460/javma.2004.225.384 15328713

[27] Payne JR Brodbelt DC Luis Fuentes V. Cardiomyopathy prevalence in 780 apparently healthy cats in rehoming centres (the CatScan study). J Vet Cardiol. (2015) 17:S244–57. doi: 10.1016/j.jvc.2015.03.008 26776583

[28] Ferasin L Ferasin H Cala A Creelman N. Prevalence and clinical significance of heart murmurs detected on cardiac auscultation in 856 cats. Vet Sci. (2022) 9:564. doi: 10.3390/vetsci9100564 36288177 PMC9611806

[29] Carney HC Ward CR Bailey SJ Bruyette D Dennis S Ferguson D . 2016 AAFP guidelines for the management of feline hyperthyroidism. J Feline Med Surg. (2016) 18:400–16. doi: 10.1177/1098612X16643252 27143042 PMC11132203

[30] Sparkes AH Cannon M Church D Fleeman L Harvey A Hoenig M . ISFM consensus guidelines on the practical management of diabetes mellitus in cats. J Feline Med Surg. (2015) 17:235–50. doi: 10.1177/1098612X15571880 25701862 PMC11148891

[31] Dagher E Abadie J Loussouarn D Campone M Nguyen F. Feline invasive mammary carcinomas: prognostic value of histological grading. Vet Pathol. (2019) 56:660–70. doi: 10.1177/0300985819846870 31113336

[32] Lascelles BDX Dong Y-H Marcellin-Little DJ Thomson A Wheeler S Correa M. Relationship of orthopedic examination, goniometric measurements, and radiographic signs of degenerative joint disease in cats. BMC Vet Res. (2012) 8:10. doi: 10.1186/1746-6148-8-10 22281125 PMC3293090

[33] Dowgray N Comerford E German AJ Gardiner J Pinchbeck G Bates KT. Paw pressure and gait in middle-aged client-owned cats with and without naturally-occurring musculoskeletal disease. PLoS ONE. (2024) 19:e0314629. doi: 10.1371/journal.pone.0314629 39693355 PMC11654939

[34] R Core Team. R: A Language and Environment for Statistical Computing. Vienna: R Foundation for Statistical Computing (2024).

[35] Wickham H François R Henry L Müller K Vaughan D. dplyr: a grammar of data manipulation. R package version 1.1. 2. Computer software (2023).

[36] Wickham H. ggplot2: Elegant Graphics for Data Analysis. New York, NY: Springer-Verlag New York. (2016).

[37] Lüdecke D. sjPlot: Data Visualization for Statistics in Social Science. R package version 2.8.16 ed. (2024).

[38] Sjoberg DD Whiting K Curry M Lavery JA Larmarange J. Reproducible summary tables with the gtsummary package. R J. (2021) 13:570–80. doi: 10.32614/RJ-2021-053

[39] Wickham H Henry L. purrr: Functional Programming Tools. R package version 1.0.4 ed.) (2025).

[40] Newcombe RG. Two-sided confidence intervals for the single proportion: comparison of seven methods. Stat Med. (1998) 17:857–72. doi: 10.1002/(sici)1097-0258(19980430)17:8<857::aid-sim777>3.0.co;2-e 9595616

[41] Bates D Mächler M Bolker B Walker S. Fitting linear mixed-effects models using lme4. J. Stat. Softw. (2015) 67:1–48. doi: 10.18637/jss.v067.i01

[42] Hartig F. DHARMa: residual diagnostics for hierarchical (multi-level/mixed) regression models. CRAN: Contributed Packages. (2016).

[43] Lüdecke D Ben-Shachar MS Patil I Waggoner P Makowski D. Performance: an R package for assessment, comparison and testing of statistical models. J Open Source Software. (2021) 6. doi: 10.31234/osf.io/vtq8f

[44] Noordzij M Dekker FW Zoccali C Jager KJ. Measures of disease frequency: prevalence and incidence. Nephron Clin Prac. (2010) 115:c17–20. doi: 10.1159/000286345 20173345

[45] Therneau T Lumley T. R survival package. R Core Team. (2013) p. 523.

[46] Sjoberg DD Baillie M Haesendonckx S Treis T. ggsurvfit: flexible time-to-event figures. R package version. 0.3*:* 0. (2023).

[47] Kassambara A Kosinski M Biecek P Fabian S. Survminer: Drawing Survival Curves Using'Ggplot2'. R Package Version 0.4. 8. (2020).

[48] Harrell Jr FE Harrell Jr MFE Hmisc D. Package ‘rms'. Vanderbilt Univ. (2017) 229.

[49] Lund E. Epidemiology of periodontal disease in older cats. Vet Focus. (2012) 22:23–4. doi: 10.1055/s-0034-1381881

[50] Murray JK Casey RA Gale E Buffington CaT Roberts C Kinsman RH . Cohort profile: the ‘bristol cats study' (BCS)–a birth cohort of kittens owned by UK households. Int J Epidemiol. (2017) 46:1749–50e. doi: 10.1093/ije/dyx066 28645213

[51] Slingerland LI Hazewinkel HA Meij BP Picavet P Voorhout G. Cross-sectional study of the prevalence and clinical features of osteoarthritis in 100 cats. Vet J. (2011) 187:304–9. doi: 10.1016/j.tvjl.2009.12.014 20083417

[52] Godfrey DR. Osteoarthritis in cats: a retrospective radiological study. J Small Animal Prac. (2005) 46:425–9. doi: 10.1111/j.1748-5827.2005.tb00340.x 16167592

[53] Dowgray N Comerford E. Feline musculoskeletal ageing: how are we diagnosing and treating musculoskeletal impairment? J Feline Med Surg. (2020) 22:1069–83. doi: 10.1177/1098612X20965832 33100170 PMC10814220

[54] Rodan I Dowgray N Carney HC Carozza E Ellis SL Heath S . 2022 AAFP/ISFM cat friendly veterinary interaction guidelines: approach and handling techniques. J Feline Med Surg. (2022) 24:1093–132. doi: 10.1177/1098612X221128760 36259500 PMC10845437

[55] Enomoto M Lascelles BDX Robertson JB Gruen ME. Refinement of the Feline Musculoskeletal Pain Index (FMPI) and development of the short-form FMPI. J Feline Med Surg. (2022) 24:142–51. doi: 10.1177/1098612X211011984 34002643 PMC10812168

[56] Guillot M Moreau M Heit M Martel-Pelletier J Pelletier JP Troncy E. Characterization of osteoarthritis in cats and meloxicam efficacy using objective chronic pain evaluation tools. Vet J. (2013) 196:360–7. doi: 10.1016/j.tvjl.2013.01.009 23416029

[57] Gruen ME Alfaro-Córdoba M Thomson AE Worth AC Staicu AM Lascelles BD. The use of functional data analysis to evaluate activity in a spontaneous model of degenerative joint disease associated pain in cats. PLoS ONE. (2017) 12:e0169576. doi: 10.1371/journal.pone.0169576 28099449 PMC5242440

[58] Cruz-Jentoft AJ Bahat G Bauer J Boirie Y Bruyère O Cederholm T . Sarcopenia: revised European consensus on definition and diagnosis. Age Ageing. (2019) 48:16–31. doi: 10.1093/ageing/afy169 30312372 PMC6322506

[59] Wang H Bai J He B Hu X Liu D. Osteoarthritis and the risk of cardiovascular disease: a meta-analysis of observational studies. Sci Rep. (2016) 6:39672. doi: 10.1038/srep39672 28004796 PMC5177921

[60] Hall AJ Stubbs B Mamas MA Myint PK Smith TO. Association between osteoarthritis and cardiovascular disease: systematic review and meta-analysis. Eur J Prev Cardiol. (2020) 23:938–46. doi: 10.1177/2047487315610663 26464295

[61] Ramasamy SK Kusumbe AP Schiller M Zeuschner D Bixel MG Milia C . Blood flow controls bone vascular function and osteogenesis. Nat Commun. (2016) 7:13601. doi: 10.1038/ncomms13601 27922003 PMC5150650

[62] Lo K Au M Ni J Wen C. Association between hypertension and osteoarthritis: A systematic review and meta-analysis of observational studies. J Orthopaed Transl. (2022) 32:12–20. doi: 10.1016/j.jot.2021.05.003 35591938 PMC9072802

[63] Knies M Kooistra HS Teske E. Prevalence of persistent hypertension and situational hypertension in a population of elderly cats in The Netherlands. J Fel Med Surg. (2023) 25:1098612X231172629. doi: 10.1177/1098612X231172629 37278217 PMC10811978

[64] Sparkes A Garelli-Paar C Blondel T Guillot E. ‘The mercury challenge': feline systolic blood pressure in primary care practice–a European survey. J Feline Med Surg. (2022) 24:e310–23. doi: 10.1177/1098612X221105844 35757930 PMC9511504

[65] Conroy M Brodbelt DC O'neill D Chang Y-M Elliott J. Chronic kidney disease in cats attending primary care practice in the UK: a VetCompassTM study. Vet Record. (2019) 184:526. doi: 10.1136/vr.105100 31023949

[66] Marino CL Lascelles BDX Vaden SL Gruen ME Marks SL. Prevalence and classification of chronic kidney disease in cats randomly selected from four age groups and in cats recruited for degenerative joint disease studies. J Feline Med Surg. (2014) 16:465–72. doi: 10.1177/1098612X13511446 24217707 PMC4414065

[67] Hoffman JM Creevy KE Franks A O'neill DG Promislow DEL. The companion dog as a model for human aging and mortality. Aging Cell. (2018) 17:e12737. doi: 10.1111/acel.12737 29457329 PMC5946068

[68] Kirchberger I Meisinger C Heier M Zimmermann A-K Thorand B Autenrieth CS . Patterns of multimorbidity in the aged population results from the KORA-age study. PLoS ONE. (2012) 7:e30556. doi: 10.1371/journal.pone.0030556 22291986 PMC3264590

[69] Pazan F Wehling M. Polypharmacy in older adults: a narrative review of definitions, epidemiology and consequences. Eur Geriatr Med. (2021) 12:443–52. doi: 10.1007/s41999-021-00479-3 33694123 PMC8149355

[70] Campisi J Kapahi P Lithgow GJ Melov S Newman JC Verdin E. From discoveries in ageing research to therapeutics for healthy ageing. Nature. (2019) 571:183–92. doi: 10.1038/s41586-019-1365-2 31292558 PMC7205183

[71] Anisimov VN Berstein LM Egormin PA Piskunova TS Popovich IG Zabezhinski MA . Metformin slows down aging and extends life span of female SHR mice. Cell Cycle. (2008) 7:2769–73. doi: 10.4161/cc.7.17.6625 18728386

[72] Chen C Liu Y Liu Y Zheng P. mTOR regulation and therapeutic rejuvenation of aging hematopoietic stem cells. Sci Signal. (2009) 2:ra75. doi: 10.1126/scisignal.2000559 19934433 PMC4020596

[73] Harrison DE Strong R Sharp ZD Nelson JF Astle CM Flurkey K . Rapamycin fed late in life extends lifespan in genetically heterogeneous mice. Nature. (2009) 460:392–5. doi: 10.1038/nature08221 19587680 PMC2786175

[74] Anisimov VN Zabezhinski MA Popovich IG Piskunova TS Semenchenko AV Tyndyk ML . Rapamycin extends maximal lifespan in cancer-prone mice. Am J Pathol. (2010) 176:2092–7. doi: 10.2353/ajpath.2010.091050 20363920 PMC2861075

[75] Martin-Montalvo A Mercken EM Mitchell SJ Palacios HH Mote PL Scheibye-Knudsen M . Metformin improves healthspan and lifespan in mice. Nat Commun. (2013) 4:2192. doi: 10.1038/ncomms3192 23900241 PMC3736576

[76] Coleman AE Creevy KE Anderson R Reed MJ Fajt VR Aicher KM . Test of Rapamycin in Aging Dogs (TRIAD): study design and rationale for a prospective, parallel-group, double-masked, randomized, placebo-controlled, multicenter trial of rapamycin in healthy middle-aged dogs from the Dog Aging Project. Geroscience. (2025) 47:2851–77. doi: 10.1007/s11357-024-01484-7 39951177 PMC12181551

[77] Kaplan JL Rivas VN Walker AL Grubb L Farrell A Fitzgerald S . Delayed-release rapamycin halts progression of left ventricular hypertrophy in subclinical feline hypertrophic cardiomyopathy: results of the RAPACAT trial. J Am Vet Med Assoc. (2023) 261:1628–37. doi: 10.2460/javma.23.04.0187 37495229 PMC10979416

[78] North Carolina State University College of Veterinary Medicine. A blinded, randomized, and placebo-controlled clinical trial of a novel veterinary product for the management of chronic kidney disease in cats. [Online]. Raleigh, NC: NC State University College of Veterinary Medicine. (2024). Available online at: https://cvm.ncsu.edu/a-blinded-randomized-and-placebo-controlled-clinical-trial-of-a-novel-veterinary-product-for-the-management-of-chronic-kidney-disease-in-cats/ [Accessed November 10, 2025].

[79] Dakic T Jevdjovic T Vujovic P Mladenovic A. The less we eat, the longer we live: can caloric restriction help us become centenarians? Int J Mol Sci. (2022) 23. doi: 10.3390/ijms23126546 35742989 PMC9223351

[80] Varela-López A Romero-Márquez JM Navarro-Hortal MD Ramirez-Tortosa CL Battino M Forbes-Hernández TY . Dietary antioxidants and lifespan: relevance of environmental conditions, diet, and genotype of experimental models. Exp Gerontol. (2023) 178:112221. doi: 10.1016/j.exger.2023.112221 37230336

[81] Fabian P Blander G Deehan R Torkamani A Nogal B. Causal impact of genetically-determined fish and fish oil intake on epigenetic age acceleration and related serum markers. Hum Genomics. (2025) 19:61. doi: 10.1186/s40246-025-00756-3 40410862 PMC12102828

[82] Cupp C Jean-Philippe C Kerr W Patil A Perez-Camargo G. Effect of nutritional interventions on longevity of senior cats. Int J Appl Res Vet Med. (2006) 4:34–50.

[83] Teng KT Mcgreevy PD Toribio J-aL Raubenheimer D Kendall K Dhand NK. Strong associations of nine-point body condition scoring with survival and lifespan in cats. J Feline Med Surg. (2018) 20:1110–8. doi: 10.1177/1098612X17752198 29393723 PMC11104206

[84] Montoya M Morrison JA Arrignon F Spofford N Charles H Hours M-A . Life expectancy tables for dogs and cats derived from clinical data. Frontiers in Veterinary Science. (2023) 10. doi: 10.3389/fvets.2023.1082102 36896289 PMC9989186

[85] Teng KT-Y Brodbelt DC Church DB O'neill DG. Life tables of annual life expectancy and risk factors for mortality in cats in the UK. J Feline Med Surg. (2024) 26:1098612X241234556. doi: 10.1177/1098612X241234556 38714312 PMC11156239

[86] Aune D Sen A Prasad M Norat T Janszky I Tonstad S . BMI and all cause mortality: systematic review and non-linear dose-response meta-analysis of 230 cohort studies with 374 million deaths among 303 million participants. BMJ. (2016). 353:2156. doi: 10.1136/bmj.i2156 27146380 PMC4856854

[87] Freeman LM. Cachexia and sarcopenia: emerging syndromes of importance in dogs and cats. J Vet Intern Med. (2012) 26:3–17. doi: 10.1111/j.1939-1676.2011.00838.x 22111652

[88] Hua J Hoummady S Muller C Pouchelon J-L Blondot M Gilbert C . Assessment of frailty in aged dogs. Am J Vet Res. (2016) 77:1357–65. doi: 10.2460/ajvr.77.12.1357 27901392

[89] Martin FC Ranhoff AH. Frailty and sarcopenia, in *Orthogeriatrics: The Management of Older Patients with Fragility Fractures*. eds. P. Falaschi and D. Marsh. Cham: Springer. (2021) p. 53–65. 33347100

[90] Russell KJ Mondino A Fefer G Griffith E Saker K Gruen ME .. Establishing a clinically applicable frailty phenotype screening tool for aging dogs. Front Vet Sci. (2024) 11:1335463. doi: 10.3389/fvets.2024.1335463 39391218 PMC11465091

[91] Colleran EJ Delgado MM Ren Y German AJ Gruen ME Gunn-Moore DA . A non-randomized pilot study to test the feasibility of developing a frailty scale for pet cats. Front Vet Sci. (2025) 12:1549566. doi: 10.3389/fvets.2025.1549566 40078212 PMC11897749

[92] Matsumoto Y Ruamrungsri N Arahori M Ukawa H Ohashi K Lyons LA . Genetic relationships and inbreeding levels among geographically distant populations of *Felis catus* from Japan and the United States. Genomics. (2021) 113:104–10. doi: 10.1016/j.ygeno.2020.11.018 33246017

[93] Toribio JA Norris JM White JD Dhand NK Hamilton SA Malik R. Demographics and husbandry of pet cats living in Sydney, Australia: results of cross-sectional survey of pet ownership. J Feline Med Surg. (2009) 11:449–61. doi: 10.1016/j.jfms.2008.06.010 19070524 PMC7130031

[94] Paynter AN Dunbar MD Creevy KE Ruple A. Veterinary big data: when data goes to the dogs. Animals (Basel). (2021) 11. doi: 10.3390/ani11071872 34201681 PMC8300140

[95] Cats Protection. Cats and their stats (CATS) report 2024. East Sussex:Cats Protection (2024).

[96] Pye CR. Physical, Biochemical, Haematological and Metabolomic Biomarkers of Ageing in Cats: Findings from the Cat Prospective Ageing and Welfare Study. (thesis). Liverpool: University of Liverpool. (2025).

[97] O'Neill DG Gunn-Moore D Sorrell S Mcauslan H Church DB Pegram C Brodbelt DC. Commonly diagnosed disorders in domestic cats in the UK and their associations with sex and age. J Feline Med Surg. (2023) 25:1098612X231155016. doi: 10.1177/1098612X231155016 36852509 PMC10812063

[98] International Renal Interest Society. IRIS Staging of CKD (modified 2019). (2019). Available online at: http://www.iris-kidney.com/pdf/IRIS_Staging_of_CKD_modified_2019.pdf (Accessed February 17, 2022).

[99] Wei T Simko V Levy M Xie Y Jin Y Zemla J. Corrplot: Visualization of a correlation matrix (0.84) [computer software]. (2021). Available online at: https://CRAN.R-project.org/package=corrplot

