# Supplementary material for: Patterns of morbidity, multimorbidity, and mortality in aging cats: findings from seven years of the Cat Prospective Ageing and Welfare Study
Source: Front Vet Sci. 2026 Jun 24;13:1813450. doi: 10.3389/fvets.2026.1813450 (PMC13347123; doi:10.3389/fvets.2026.1813450)
Supplement: Supplementary file 1 [file Data_Sheet_1.pdf]

Supplementary Material

1 Supplementary Figures

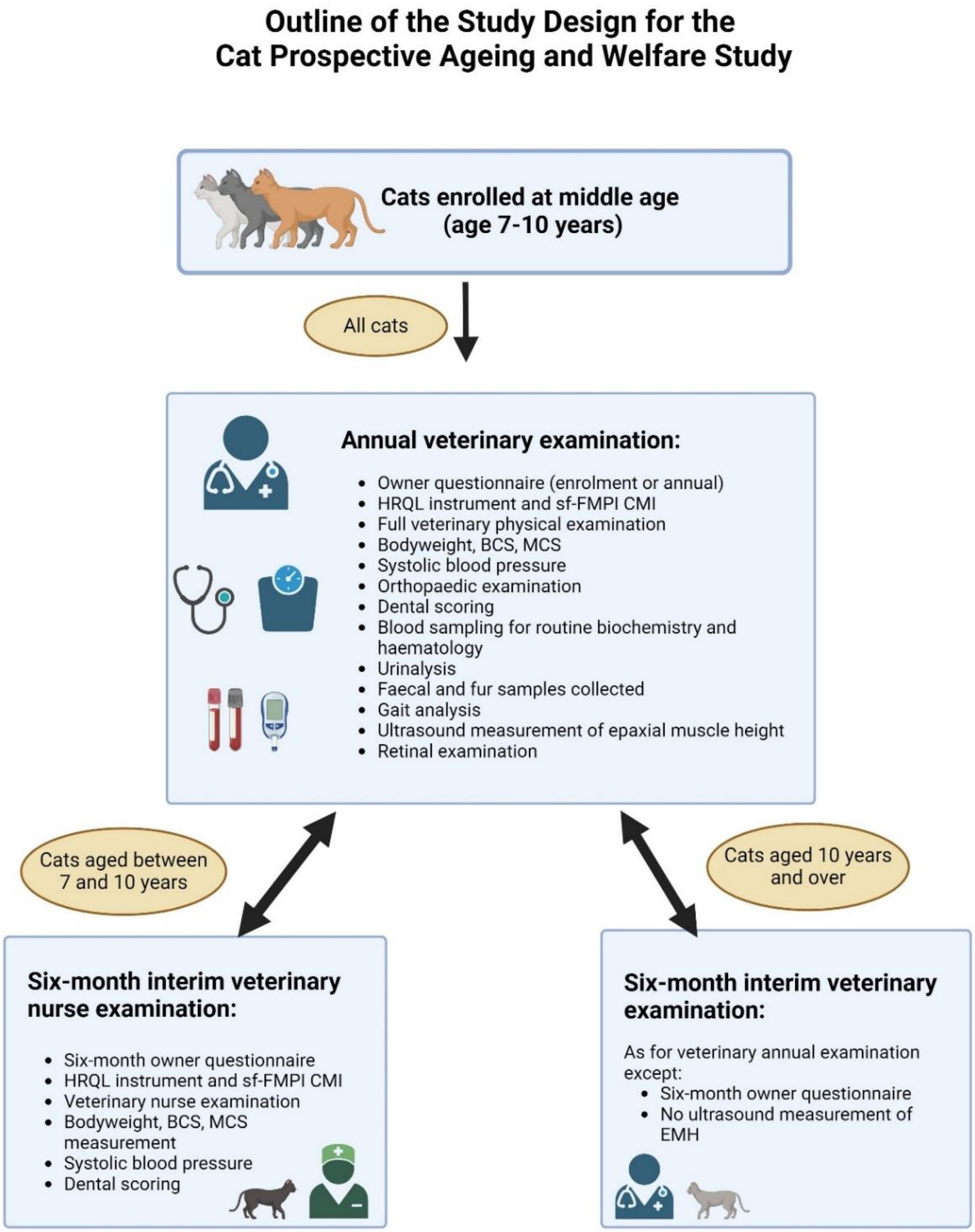

Supplementary Figure 1. Schematic outline of the Cat Prospective Ageing and Welfare Study. Abbreviations: HRQL= healthy related quality of life; sf-FMPI CMI = short form Feline Musculoskeletal Pain Index clinical metrology instrument; BCS= body condition score; MCS = muscle condition score; EMH= epaxial muscle height

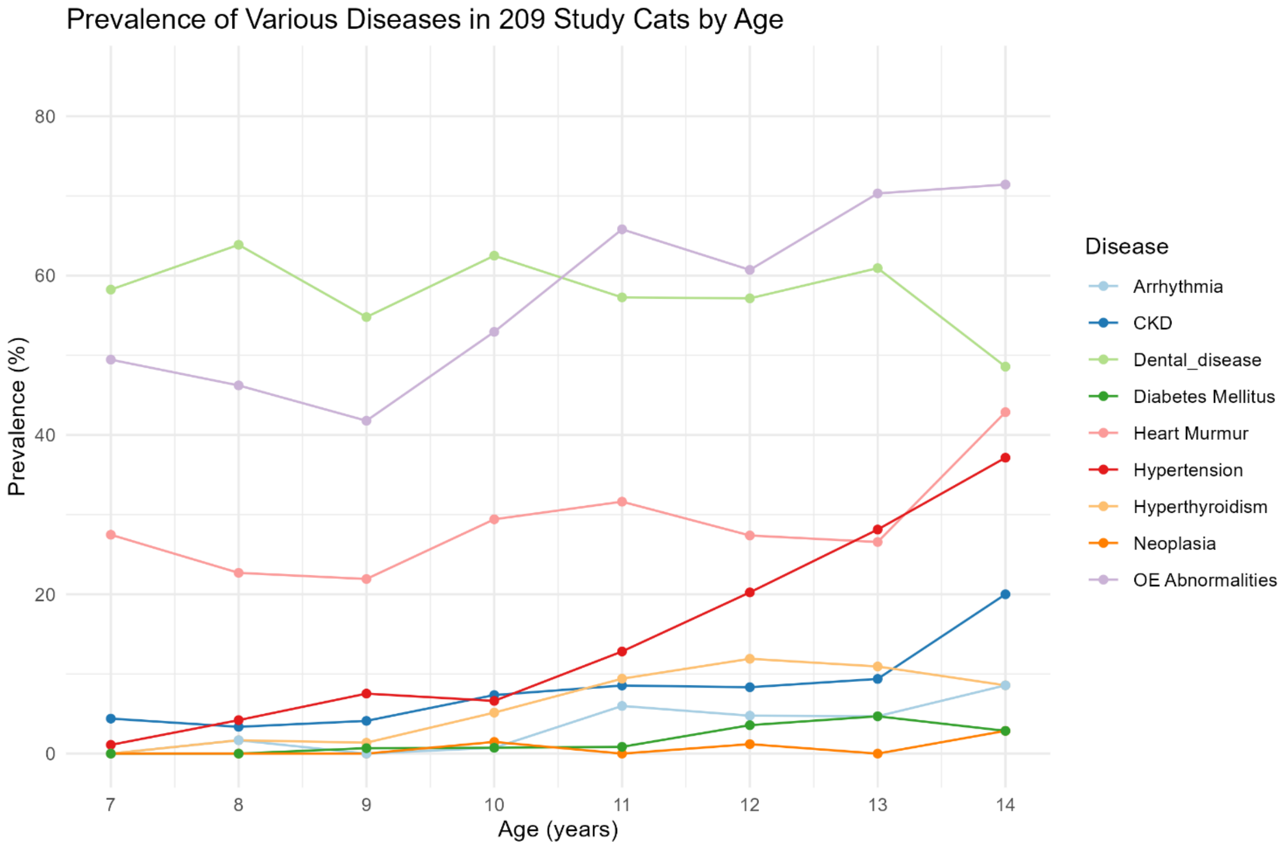

Suplementary Figure 2. Descriptive prevalences of different age-related diseases at 7 to 14 years of age in 209 cats enrolled on the Cat Prospective Ageing and Welfare Study between February 2017 and January 2024. CKD=chronic kidney disease, OE=orthopaedic examination. Number of cats at each year of age were as follows: age 7=91; age 8=119; age 9=146; age 10=136; age 11=117; age 12=88; age 13=66; age 14=35;. NB- if a cat had more than one veterinary examination within the year, only data from one examination (prioritising the visit with diagnosis of disease) was included to avoid duplicates of the same cat per year group. These estimates are descriptive and do not account for repeated measures contributed by individual cats across multiple age groups.

frontiers

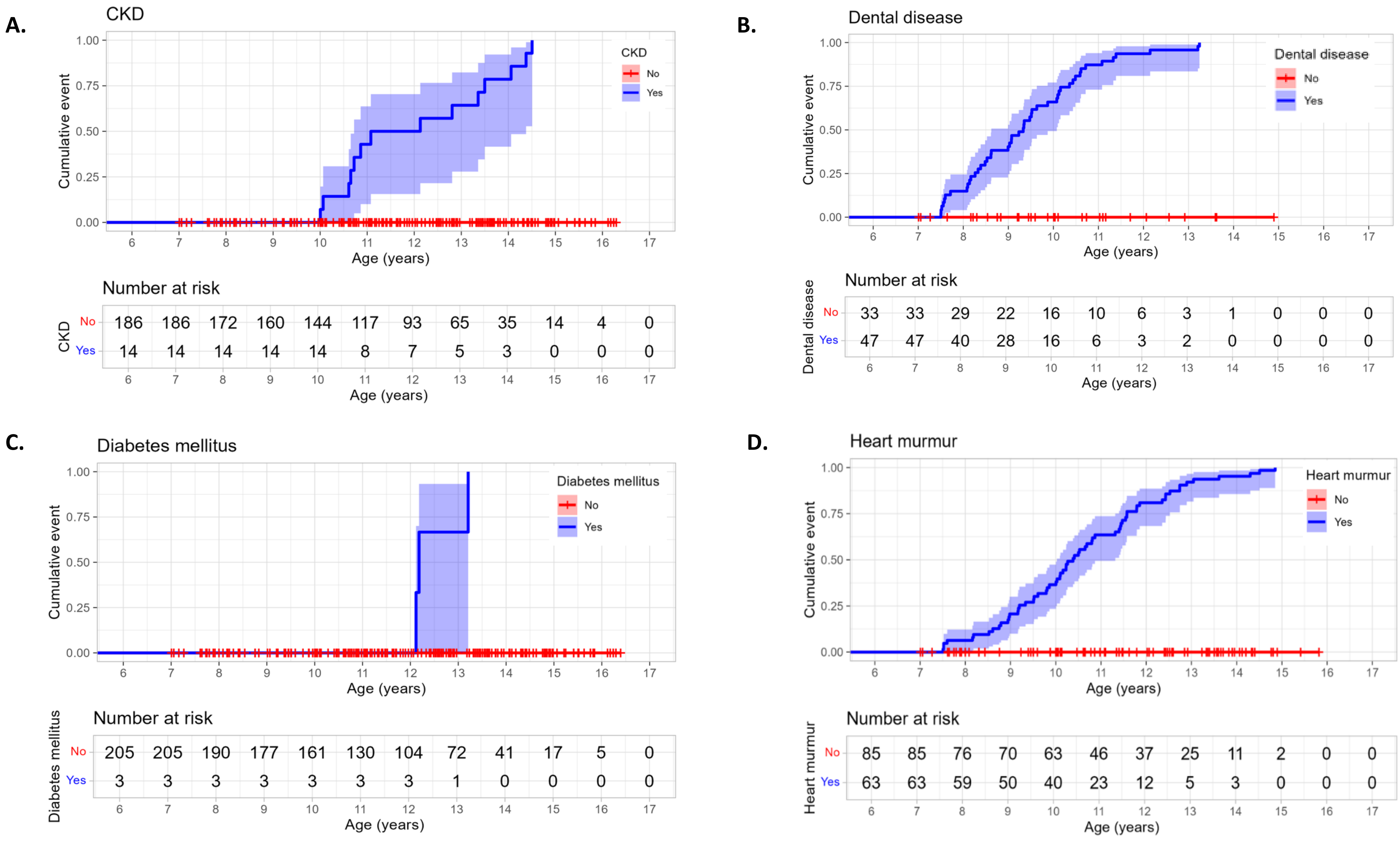

Supplementary Figure 3. (continues overleaf)

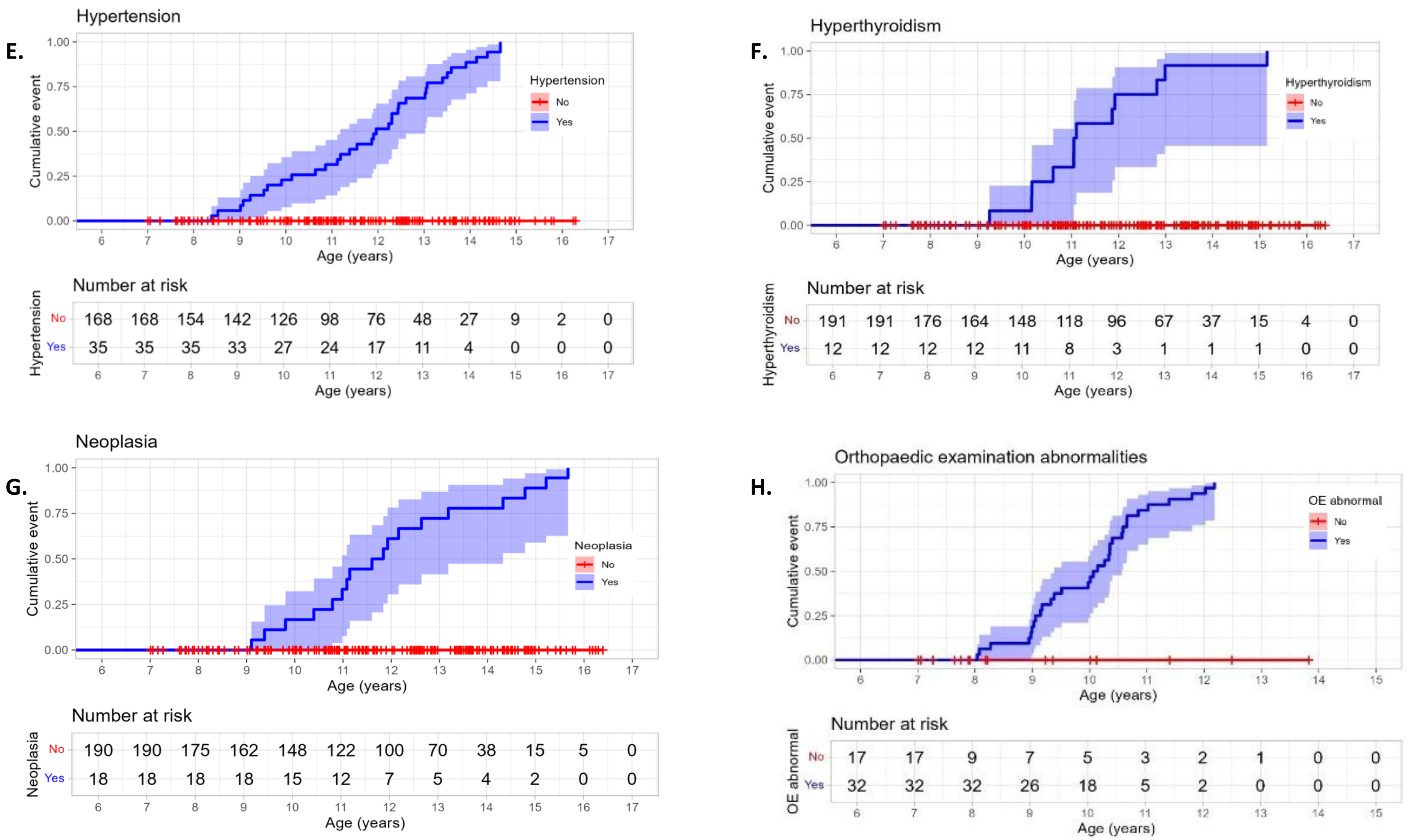

Supplementary Figure 3 (continued). Age at diagnosis of A) Chronic kidney disease (CKD), B) Dental disease, C) Diabetes mellitus, D) Heart murmurs, E) Hypertension, F) Hyperthyroidism, G) Neoplasia and H) Orthopaedic examination abnormalities in cats enrolled on the Cat Prospective Ageing and Welfare Study between February 2017 and January 2024. Pre-existing diagnoses or diagnoses made at enrolment examination were excluded from each disease analysis. Crosses denote censored data (the age at loss to follow up or at the end of study period). The "number at risk" table indicates the number of individuals still under observation and event free at the start of each age-year, with "No" indicating subjects that did not develop the condition and "Yes" indicating subjects that did eventually develop the condition.

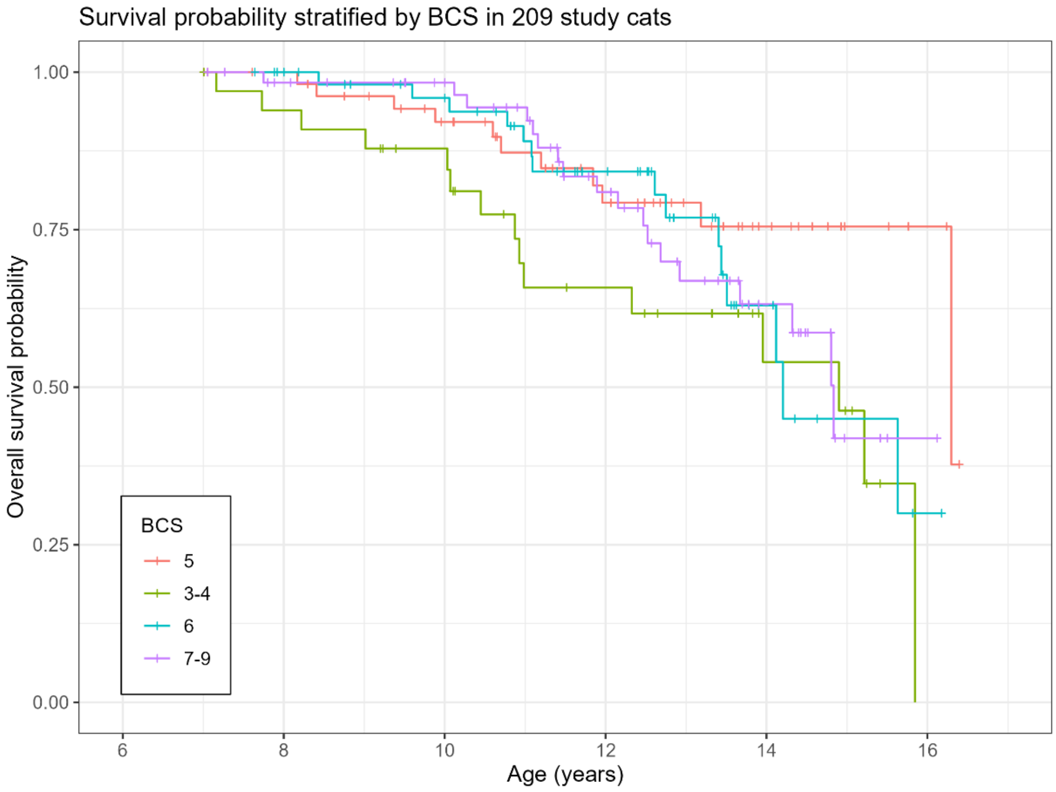

Supplementary Figure 4. Kaplan Meier curve showing survival in 209 study cats stratified by body condition score (BCS) at enrolment. No cat had a BCS of <3 at enrolment.

2 Supplementary Tables

Supplementary Table 1. A summary of signalment information for the 209 study cats.

Variable		Baseline value at enrolment
**Sex (N, %)**	Female	111 (53%)
	Male	98 (47%)
**Breed (N, %)**		
	DLH	23 (11%)
	DMH	24 (11%)
	DSH	133 (64%)
	**Total**	**180 (86%)**
	American Exotic	2 (1.0%)
	Balinese	1 (0.5%)
	Bengal	3 (1.4%)
	British short hair	3 (1.4%)
	Egyptian Mau	1 (0.5%)
	Maine coon	2 (1.0%)
	Ocicat	1 (0.5%)
	Oriental	2 (1.0%)
	Persian	4 (1.9%)
	Ragdoll	4 (1.9%)
	Russian blue	1 (0.5%)
	Siamese	3 (1.4%)
	Somali	1 (0.5%)
	Sphynx	1 (0.5%)
	**Total**	**29 (14%)**
**Age (in years; mean [SD])**		8.3 (1.11)
**Bodyweight (kg; mean [SD]))**		4.8(1.10)
**SBP (mmHg; mean [SD])**		128 (18.0)
**BCS (1-9 scale; median [IQR])**		6 (2)
**MCS (combined score 0-30; median [IQR])**		30 (2)

Abbreviations: DSH=Domestic shorthair, DMH=Domestic medium hair, DLH=Domestic long hair, SBP=Systolic blood pressure, BCS=Body condition score, MCS = muscle condition score, SD= standard deviation, IQR=interquartile range.

Supplementary Table 2. Number of veterinary examinations conducted for the 209 study cats.

Number of visits per cat	Number of cats
**1**	29
**2**	21
**3**	14
**4**	19
**5**	18
**6**	12
**7**	18
**8**	11
**9**	24
**10**	19
**11**	18
**12**	6
**Total number of cats**	209
**Total number of visits**	1241

NB: Visits to the Feline Healthy Ageing Clinic include the enrolment visit and subsequent veterinary and veterinary nurse examinations at any timepoint during the study period and so do not represent sequential visits. For example, cats with three visits may have had gaps between visits of varying time.

Supplementary Table 3. Reasons for discontinued participation for 59 study cats.

**Cause of drop-out from study**	**Number of cats**	**%**
Owner not responding to communication from clinic staff	28	47.5
Owner moved address	9	15.3
Owner withdrew as cat too stressed	7	11.9
Owner withdrew as too busy	5	8.5
Owner withdrew as could not travel to clinic	3	5.1
Cat went missing	2	3.4
Owner withdrew due to ill health	2	3.4
Cat rehomed	1	1.7
Owner died and cat rehomed	1	1.7
Owner unwilling to visit practice hosting the FHAC after death of their other cat during a procedure at that practice.	1	1.7
**Total**	**59**	**100.0**

Abbreviations: FHAC= feline healthy ageing clinic.

Supplementary Table 4. Results of binomial mixed-effects logistic regression models investigating the association of age, sex and their interactions on the predicted probability of various diseases within the study cats.

	Dental disease			Hypertension			Heart murmur			OE abnormalities		
Predictors	*Odds Ratios*	*CI*	*p*	*Odds Ratios*	*CI*	*p*	*Odds Ratios*	*CI*	*p*	*Odds Ratios*	*CI*	*p*
(Intercept)	1.33	0.58 – 3.07	0.496	0.00	0.00 – 0.01	**<0.001**	0.13	0.04 – 0.42	**0.001**	0.46	0.20 – 1.08	0.075
Age years [1st degree]	0.94	0.39 – 2.27	0.888	19.57	2.65 – 144.33	**0.004**	2.66	0.84 – 8.38	0.095	2.64	1.03 – 6.77	**0.044**
Age years [2nd degree]	0.66	0.08 – 5.34	0.701	661.73	1.59 – 275876.25	**0.035**	5.42	0.36 – 81.94	0.222	14.11	1.49 – 133.40	**0.021**
Age years [3rd degree]	0.49	0.11 – 2.22	0.359	167.85	9.52 – 2960.33	**<0.001**	17.51	2.86 – 107.06	**0.002**	12.11	2.19 – 67.08	**0.004**
Sex [Male]	0.64	0.19 – 2.18	0.474	0.45	0.01 – 29.05	0.709	1.18	0.23 – 6.00	0.845	1.13	0.32 – 4.00	0.849
Age years [1st degree] × Sex [Male]	1.71	0.49 – 5.95	0.402	19.88	0.85 – 464.47	0.063	0.74	0.16 – 3.48	0.701	4.97	1.30 – 18.95	**0.019**
Age years [2nd degree] × Sex [Male]	6.30	0.31 – 128.04	0.231	205.07	0.03 – 1340489.37	0.235	1.24	0.03 – 59.04	0.915	0.61	0.02 – 15.23	0.765
Age years [3rd degree] × Sex [Male]	3.88	0.49 – 30.80	0.200	51.19	0.94 – 2776.83	0.053	0.30	0.03 – 3.52	0.336	0.86	0.08 – 8.95	0.903
**Random Effects**												
σ^2	3.29			3.29			3.29			3.29		
τ_{00}	1.51 $_{Cat\_ID}$			12.16 $_{Cat\_ID}$			3.18 $_{Cat\_ID}$			1.34 $_{Cat\_ID}$		
ICC	0.32			0.79			0.49			0.29		
N	208 $_{Cat\_ID}$			204 $_{Cat\_ID}$			209 $_{Cat\_ID}$			209 $_{Cat\_ID}$		
Observations	1206			1172			1228			1151		
Marginal R^2 / Conditional R^2	0.008 / 0.320			0.211 / 0.832			0.045 / 0.514			0.063 / 0.360		

Abbreviations: OE=Orthopaedic examination. Age years 1st to 3rd degrees relate to 3 spline coefficients for a natural cubic spline with 3 degrees of freedom. P-values shown in bold denote statistical significance (P<0.05).

Supplementary Table 5. Combinations of concurrent morbidities that occurred in the 209 study cats.

Number of morbidities	Morbidities present	Number of cats
1	Dental disease	13
	OE abnormality	8
	Heart murmur	3
	Neoplasia	1
	**Total**	**25**
2	Dental disease, OE abnormalities	28
	Heart murmur, OE abnormality	9
	Heart murmur, Dental disease	4
	Hypertension, Dental disease	1
	Hypertension, OE abnormality	1
	Neoplasia, Dental disease	1
	**Total**	**44**
3	Heart murmur, dental disease, OE abnormality	55
	Hypertension, dental disease, OE abnormality	7
	Neoplasia, dental disease, OE abnormality	4
	CKD, dental disease, OE abnormality	4
	Heart murmur, hypertension, hyperthyroidism	1
	Heart murmur, hypertension, hyperthyroidism	1
	Heart murmur, hyperthyroidism, dental disease	1
	Heart murmur, diabetes mellitus, dental disease	1
	Heart murmur, neoplasia, OE abnormality	1
	Hypertension, CKD, dental disease	1
	Hyperthyroid, dental disease, OE abnormality	1
	Diabetes mellitus, dental disease, OE abnormality	1
	**Total**	**78**
4	Heart murmur, hypertension, dental disease, OE abnormality	14
	Heart murmur, CKD, dental disease, OE abnormality	8
	Heart murmur, neoplasia, dental disease, OE abnormality	7

	Heart murmur, hyperthyroidism, dental disease, OE abnormality	6
	Hypertension, CKD, dental disease, OE abnormality	2
	Heart murmur, diabetes mellitus, dental disease, OE abnormality	1
	Hyperthyroidism, CKD, dental disease, OE abnormality	1
	CKD, neoplasia, dental disease, OE abnormality	1
	**Total**	**40**
**5**	Heart murmur, hypertension, hyperthyroidism, dental disease, OE abnormality	5
	Heart murmur, hypertension, neoplasia, dental disease, OE abnormality	2
	Heart murmur, hypertension, CKD, dental disease, OE abnormality	1
	**Total**	**8**
**6**	Heart murmur, hypertension, CKD, dental disease, OE abnormality, hyperthyroidism	2
	Heart murmur, hypertension, CKD, dental disease, OE abnormality, diabetes	1
	Heart murmur, hypertension, CKD, dental disease, OE abnormality, neoplasia	1
	**Total**	**4**

Abbreviations: OE=Orthopaedic examination; CKD = chronic kidney disease.

Supplementary Table 6. The five top-performing Cox Proportional Hazards Models investigating baseline factors associated with mortality in study cats and a comparison of their Bayesian Information Criteria.

Model	BIC
Surv(survival_time_years, survival_status) ~ Breed + MCS_grade	469.1390
Surv(survival_time_years, survival_status) ~ Breed	470.3855
Surv(survival_time_years, survival_status) ~ Breed + Hyperthyroidism	470.9913
Surv(survival_time_years, survival_status) ~ Breed + MCS_grade + Hyperthyroidism	471.4086
Surv(survival_time_years, survival_status) ~ Breed + MCS_grade + CKD	471.8041
